# Mapping human mobility during the third and second millennia BC in present-day Denmark

**DOI:** 10.1371/journal.pone.0219850

**Published:** 2019-08-21

**Authors:** Karin Margarita Frei, Sophie Bergerbrant, Karl-Göran Sjögren, Marie Louise Jørkov, Niels Lynnerup, Lise Harvig, Morten E. Allentoft, Martin Sikora, T. Douglas Price, Robert Frei, Kristian Kristiansen

**Affiliations:** 1 National Museum of Denmark, Department of Research, Collections and Conservation, Environmental Archaeology and Material Science, I.C. Modewegsvej, Brede, Kongens Lyngby, Denmark; 2 Institute for Historical Studies, University of Gothenburg, Gothenburg, Sweden; 3 Department of Forensic Medicine, University of Copenhagen, Copenhagen, Denmark; 4 Department of Archaeological Science and Conservation, Højbjerg, Moesgaard Museum, Denmark; 5 Centre for GeoGenetics, Natural History Museum of Denmark, University of Copenhagen, Copenhagen, Denmark; 6 Laboratory for Archaeological Chemistry, University of Wisconsin-Madison, Madison, WI, United States of America; 7 Department of Geoscience and Natural Resource Management, University of Copenhagen, Copenhagen, Denmark; University at Buffalo - The State University of New York, UNITED STATES

## Abstract

We present results of the largest multidisciplinary human mobility investigation to date of skeletal remains from present-day Denmark encompassing the 3^rd^ and 2^nd^ millennia BC. Through a multi-analytical approach based on 88 individuals from 37 different archaeological localities in which we combine strontium isotope and radiocarbon analyses together with anthropological investigations, we explore whether there are significant changes in human mobility patterns during this period. Overall, our data suggest that mobility of people seems to have been continuous throughout the 3^rd^ and 2^nd^ millennia BC. However, our data also indicate a clear shift in mobility patterns from around 1600 BC onwards, with a larger variation in the geographical origin of the migrants, and potentially including more distant regions. This shift occurred during a transition period at the beginning of the Nordic Bronze Age at a time when society flourished, expanded and experienced an unprecedented economic growth, suggesting that these aspects were closely related.

## Introduction

In this study, we wish to trace mobility patterns during the 3^rd^ and 2^nd^ millennia BC in the region covered by present-day Denmark, in order to ascertain if there were significant changes linked to the introduction of the metal economy after 2000 BC. The 3^rd^ millennium BC stands out as a period of migrations in western Eurasia, as pastoral steppe populations settled in temperate Europe after 2800 BC e.g. [1, 2]. This was also a period of cultural and genetic admixture e.g. [3]. From 1600 BC onwards, southern Scandinavia became more closely linked to the existing European metal trade networks [4], and from 1500 BC onwards, a period of unparalleled creativity resulted in the formation of a Nordic Bronze Age style, based on stylistic influences from Mycenean and central European workshops [5]. This signaled the beginning of a period of unprecedented burial wealth between 1500–1100 BC when c. 50.000 barrows were constructed in present-day Denmark alone [6]. More than 2000 swords are known from excavated burials, and as they constitute around 10% of the total number of burials, this suggests that a much larger number of swords could have been deposited [7]. There are more Bronze Age swords in present-day Denmark than anywhere else in Europe [8]. During this period, Denmark became Europe’s richest region with respect to number and density of metal depositions [9, 10]. However, this regional development was entirely dependent on the functioning of the long-distance metal trade as revealed by studies on the potential origin of copper [11, 12]. There are no native base metal ores in present-day Denmark. Additionally, recent investigations suggest that wool, too, was traded during the Nordic Bronze Age [13], and that a number of glass beads found as grave goods came from as far away as Mesopotamia and Egypt [14].

However, in order to understand the demographics and socio-economic dynamics of this formative period, it is essential to consider questions such as: What proportion of the population moved? Was mobility common for everyone or limited to a certain subset of people? Finally, are there significant shifts in mobility patterns over time?

Our study aims to shed light on these fundamental questions, and we present results of the largest strontium isotope investigation to date of human remains from present-day Denmark, including data from 88 individuals combined with radiocarbon analyses of 78 of these individuals. Samples were obtained from 37 different localities, and comprise diverse burial types that include adult males and females of various ages as well as children. Additionally, we conducted physical anthropological examinations of these individuals in order to determine their sex, age and potential injuries, wounds or illnesses, as well as to try to detect similarities due to kinship.

## Materials and methods

### The burials

**Fig 1:**
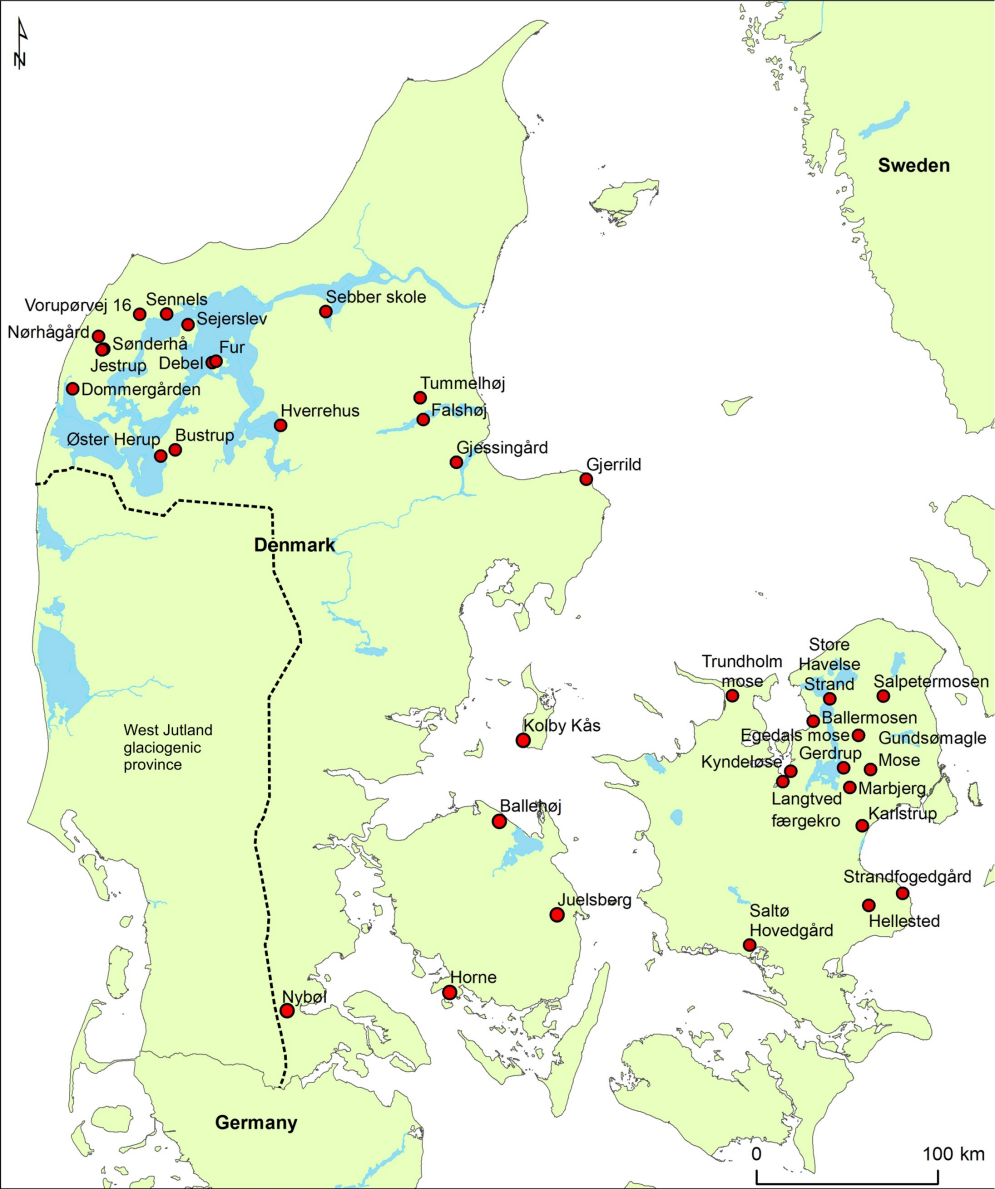
Map of present-day Denmark illustrating locations of the burial sites. The dashed black line marks the maximum advance stage of the last glaciation (Weichselian). Drafted with public domain data from Natural Earth (https://www.naturalearthdata.com).

The burials studied herein cover a wide range of grave types: gallery graves, other megalithic tombs, burial mounds (including oak coffins), and bog finds as well as flat graves. Geographically, they cover a large part of present-day Denmark (Fig 1). However, there are two areas with a higher concentration of sampled individuals, due to the more favorable soil conditions for bone preservation here. These are the island of Zealand and the area around the Limfjorden in northern Jutland, including the key area of Thy, an area known for its high density of burial mounds and numerous metal artefact finds [15]. The burials and their contexts are described in the supplementary information, including information of name, sample number, geographical location and archaeological excavation (S1 File).

### Strontium isotope analyses

Strontium isotope analyses conducted on archaeological human remains (e.g. on tooth enamel and/or cremated bone) can provide information on provenance and potential mobility at the individual level [16–18]. The strontium uptake in humans appears to be dominated by the intake of plants and water, while animal meat sources seem to play a comparatively negligible role [17, 19].

The sampling strategy at each site was based on an assessment of the archaeological context and the state of preservation of the human remains in combination. For the present study we sampled teeth of 88 individuals. We aimed at sampling first molars which mineralize early in childhood (from in utero to c. 3 years of age), but in cases where this was not possible, other available teeth were sampled. Some teeth could only be specified according to tooth category, e.g. whether they are molars or premolars (except for three samples whose preservation was too poor). Although the aim was to avoid third molars as these mineralize latest [20], in two cases third molars were sampled due to lack of other samples (S1 Table).

Tooth enamel samples were pre-cleaned by removing the enamel’s surface with a drill bit, and subsequetly, a few milligrams of enamel powder (or small pieces) were sampled from each tooth. The tooth enamel samples were dissolved in 7 ml Teflon beakers (Savillex) in a 1:1 solution of 0.5 ml 6 N HCl (Seastar) and 0.5 ml 30% H_2_O_2_ (Seastar). The samples typically dissolved within 5 minutes, after which the solutions were dried on a hotplate at 80°C. Subsequently, the enamel samples were taken up in a few drops of 3N HNO_3_ and then loaded onto disposable 100 μl pipette tip extraction columns into which we fitted a frit which retained a 0.2 ml stem volume of intensively pre-cleaned mesh 50–100 SrSpec (Eichrom Inc.) chromatographic resin. The elution recipe essentially followed that by [21] albeit scaled to our needs in so far as strontium was eluted / stripped by pure deionized water and then the eluate dried on a hotplate.

Thermal ionization mass spectrometry was used to determine the Sr isotope ratios. Samples were dissolved in 2.5 μl of a Ta_2_O_5_-H_3_PO_4_-HF activator solution and directly loaded onto previously outgassed 99.98% single rhenium filaments. Samples were measured at 1250–1300°C in a dynamic multi-collection mode on a VG Sector 54 IT mass spectrometer equipped with eight Faraday detectors (Institute of Geosciences and Natural Resource Management, University of Copenhagen). Five nanogram loads of the NBS 987 Sr standard that we ran during the time of the project yielded ^87^Sr/^86^Sr = 0.710238 +/- 0.000012 (n = 5, 2σ), which we compare to the generally accepted value of ^87^Sr/^86^Sr = 0.710248 [22].

#### Baseline

In order to investigate mobility and provenance by application of the strontium isotope system, it is necessary to have knowledge about the baseline or isoscape range of the isotopic composition of local bioavailable strontium [16, 17]. Several studies conducted during the last decade aimed at shedding light into the issue of how to establish baselines that can be used as reference maps for past provenance studies e.g. [23, 24–26]. However, there is as yet not a consensus to which type of proxy (e.g., surface waters, plants, soils, fauna, etc.) is the most suitable for delineating the isotopic range of bioavailable strontium signatures of an area [24]. Baselines from areas can be different depending on which type of proxy materials/archives are used to define them, and on the number of samples used for the spatial resolution. However, the efforts by many scholars aiming at constructing and better understanding baselines are visible in the recent literature. Respective studies, for example, include multi-proxy approaches applied to specific areas, such as the investigation of two Early Medieval cemeteries in Central Germany [23], the combination of multi-proxy baselines constructed through data accumulated over many years, such as recently presented for biosphere isotope domains of Great Britain [27], or baselines constructed on the basis of large numbers of samples, such as recently published from almost 1200 soil samples from all over Europe [28]. These diverse studies illustrate the complexity inherent in building suitable reference baselines for provenance studies.

The baseline for present-day Denmark has previously been characterized by strontium isotope analyses of 192 surface waters (lakes, creeks) [29, 30]. Additionally, results from supplementary baseline samples from plants, surface waters and soil extracts from different areas in present-day Denmark have since been added e.g. [31, 32, 33]. In addition, a reference map based on fauna remains has been published [34], but we are cautious with considering such fauna-based isoscapes since recent studies have mentioned the difficulties inherent with using this type of proxy e.g. [24]. Nevertheless, all the above mentioned studies are consistent in their isotope range of bioavailable strontium isotope signatures of ^87^Sr/^86^Sr = 0.708 to 0.711 (excluding the island of Bornholm). Furthermore, the Danish island of Bornholm located south of Sweden in the Baltic Sea revealed elevated bioavailable strontium isotope signatures (^87^Sr/^86^Sr > 0.711) due to the contribution of radiogenic Sr from the Precambrian basement which dominates most of the island [35]. Therefore, when we herein refer to the baseline for “present-day Denmark”, we exclude the area of Bornholm, unless otherwise mentioned.

A very recent study has questioned the suitability of baselines constructed from strontium isotopic compositions of surface waters in areas dominated by low to non-calcareous soils such as in the west Jutland glaciogenic province (Fig 1) [36]. This study suggests that due to the addition of agricultural lime to farmlands in this area, the baseline values of the surface waters are not adequate for their use in reference maps for provenance studies of past human mobility. While we appreciate and acknowledge the contribution by these authors and we respect the discussion they take up within their study, we disagree with their interpretation and conclusions. Some of the authors of the present study are currently working on a separate publication that will address and discuss this issue in detail.

In addition to the difficulties in delineating baselines, issues related to the classification of a material to be “local” to a specific area might not always be “a straightforward approach” [37]. In the study presented herein we investigated individuals that were found in areas within present-day Denmark, excluding the Danish island of Bornholm and areas of the west Jutland glaciogenic province, and we consequently use the term “local” with respect to these areal restrictions in present-day Denmark (Fig 1).

### Radiocarbon analyses

All individuals whose skeletal bone material is preserved were radiocarbon dated, with the exception of eight individuals with poor collagen preservation (Table 1). A further two samples consisted only of tooth enamel remains and could not be dated. In total, 78 individuals yielded radiocarbon dates. These samples exhibited C/N values within the accepted range for good collagen preservation, i.e. 2.9–3.6 [38]. The radiocarbon analyses were primarily performed at the Oxford Radiocarbon Accelerator Unit, University of Oxford, but a few were performed at The 14Chrono Centre, Queen’s University, Belfast. Both laboratories used their own standard protocols for collagen extraction and radiocarbon analyses. All dates were calibrated with Oxcal 4.3 using the Intcal13 calibration curve [39]. We report the radiocarbon dates according to Millard [40], and rounded up to 10 by using the calibration software.

**Table 1:** Results of radiocarbon analyses.

RISE#	Sitename	14C Lab. #	BP	1 s	calBC from	calBC to	C%	C:N
RISE 12	Sønderhå	OxA-28157	1949	23	1*	130*	41.1	3.3
RISE 14	Langtved Færgekro	OxA-28041	3550	27	-1980	-1770	43.9	3.1
RISE 15	Langtved Færgekro	OxA-28042	3527	27	-1940	-1760	43.9	3.2
RISE 16	Langtved Færgekro	OxA-28043	3442	29	-1880	-1660	42.9	3.1
RISE 17	Langtved Færgekro	OxA-28044	3488	28	-1900	-1700	43.4	3.1
RISE 18	Kolby Kås	OxA-28158	3309	26	-1650	-1510	44.6	3.1
RISE 19	Karlstrup	OxA-28160	3196	25	-1510	-1420	41.9	3.4
RISE 20	Karlstrup	OxA-28045	3162	27	-1510	-1390	42.7	3.1
RISE 20	Karlstrup	OxA-28046	3113	27	-1440	-1290	42.8	3.1
RISE 21	Karlstrup	OxA-28047	3092	29	-1430	-1280	46.5	3.1
RISE 22	Gjessinggård	OxA-28161	3053	24	-1410	-1230	43.7	3.3
RISE 23	Debel	OxA-28049	3117	26	-1450	-12980	42.2	3.2
RISE 25	Juelsberg	OxA-28050	3705	28	-2200	-2020	41.9	3.2
RISE 26	Kolby Kås	OxA-28159	3362	25	-1740	-1560	44.2	3.1
RISE 27	Juelsberg	OxA-28051	3787	30	-2340	-2060	43.2	3.2
RISE 28	Juelsberg	OxA-28052	3626	29	-2130	-1900	42.8	3.2
RISE 29	Juelsberg	OxA-28053	3653	29	-2140	-1940	43.7	3.2
RISE 30	Juelsberg	OxA-28190	3627	26	-2120	-1900	41.1	3.4
RISE 31	Juelsberg	OxA-28191	3542	26	-1960	-1770	40.2	3.3
RISE 32	Juelsberg	OxA-28192	3638	26	-2130	-1920	41.8	3.3
RISE 33	Juelsberg	OxA-28193	3731	26	-2210	-2030	41.6	3.3
RISE 36	Marbjerg	OxA-28194	3493	25	-1890	-1740	42.1	3.3
RISE 37	Marbjerg	OxA-28195	3429	26	-1880	-1650	42.2	3.3
RISE 38	Marbjerg	OxA-28196	3515	25	-1920	-1750	41.7	3.3
RISE 39	Marbjerg	OxA-28197	3727	26	-2210	-2030	41.6	3.3
RISE 40	Marbjerg	OxA-28198	3553	26	-1980	-1770	42.3	3.4
RISE 40	Marbjerg	OxA-28199	3504	26	-1900	-1740	42.6	3.3
RISE 41	Marbjerg	OxA-28224	3405	28	-1770	-1620	39.5	3.2
RISE 42	Marbjerg	OxA-28225	3681	28	-2200	-1970	43	3.1
RISE 43	Marbjerg	OxA-28226	3550	29	-2010	-1770	42.6	3.2
RISE 44	Marbjerg	OxA-28227	3522	29	-1930	-1750	42.3	3.2
RISE 45	Gerdrup	OxA-28228	3539	29	-1960	-1770	42.4	3.1
RISE 46	Gerdrup	OxA-28229	3499	29	-1910	-1740	43.2	3.2
RISE 47	Sebber skole	OxA-28258	3153	26	-1500	-1320	43.9	3.2
RISE 48	Sebber skole	OxA-28259	3156	26	-1500	-1320	42.5	3.2
RISE 49	Sebber skole	OxA-28260	3452	27	-1880	-1680	42.2	3.2
RISE 50	Sebber skole	OxA-28261	3274	26	-1620	-1500	44.1	3.2
RISE 51	Sebber skole	OxA-28288	3360	25	-1740	-1560	40.5	3.2
RISE 52	Sebber skole	OxA-28289	3342	32	-1740	-1530	40.9	3.3
RISE 53	Hellested	OxA-28290	3705	25	-2200	-2020	41.9	3.2
RISE 54	Hellested	OxA-28291	3796	25	-2300	-2140	41.4	3.2
RISE 55	Hellested	OxA-28292	3700	25	-2200	-1980	41.2	3.2
RISE 56	Hellested	OxA-28293	3689	25	-2200	-1980	40.7	3.2
RISE 57	Hellested	OxA-28294	3697	26	-2200	-1980	39.3	3.3
RISE 58	Tummelhøj	OxA-28230	3502	26	-1900	-1740	39.1	3.3
RISE 59	Tummelhøj	OxA-28231	3618	28	-2120	-1890	39.7	3.2
RISE 60	Kyndeløse	OxA-28295	3496	26	-1900	-1740	40.1	3.2
RISE 61	Kyndeløse	OxA-28296	4071	27	-2860	-2490	39.4	3.2
RISE 62	Kyndeløse	OxA-28297	4187	28	-2890	-2670	42.7	3.3
RISE 63	Kyndeløse	OxA-28262	3721	26	-2200	-2030	41.3	3.3
RISE 64	Kyndeløse	OxA-28263	4145	27	-2880	-2620	40.8	3.3
RISE 65	Kyndeløse	OxA-28264	4189	28	-2890	-2670	41.3	3.3
RISE 66	Kyndeløse	OxA-28265	3968	27	-2580	-2350	40.2	3.3
RISE 67	Sejerslev	OxA-28232	3535	27	-1950	-1770	41.5	3.2
RISE 68	Sejerslev	OxA-28233	3617	28	-2120	-1890	42.4	3.3
RISE 69	Falshøj	OxA-28266	3607	27	-2030	-1890	43.6	3.3
RISE 70	Falshøj	OxA-28267	3465	26	-1890	-1690	42.6	3.3
RISE 70	Falshøj	OxA-28268	3518	26	-1920	-1750	41.7	3.2
RISE 71	Falshöj	OxA-28269	3701	26	-2200	-2020	42.3	3.2
RISE 72	Gjerrild	OxA-28270	3410	26	-1770	-1630	42.4	3.3
RISE 76	Fur	OxA-28048	3205	28	-1530	-1420	42.1	3.2
RISE 104	Jestrup	OxA-28990	3295	29	-1640	-1500	42.6	3.3
RISE 106	Nørhågård	OxA-28991	2949	28	-1260	-1050	42.4	3.3
RISE 106	Nørhågård	OxA-28992	2943	28	-1260	-1040	41.8	3.3
RISE 166	Saltø Hovedgård	OxA-29192	3304	28	-1650	-1500	41.3	3.2
RISE 167	Saltø Hovedgård	OxA-28993	3220	29	-1610	-1420	45.6	3.3
RISE 168	Saltø Hovedgård	OxA-28994	3210	32	-1600	-1410	43.9	3.3
RISE 169	Store Havelse Strand	OxA-28995	3301	28	-1640	-1500	43.1	3.3
RISE 273	Gundsømagle Mose	OxA-30684	4578	28	-3500	-3110	42.3	3.2
RISE 274	Egedal mose	OxA-30483	2068	25	-180	-1	44	3.2
RISE 275	Salpetermosen	OxA-30484	3751	29	-2290	-2030	43.6	3.2
RISE 276	Trundholm mose	OxA-30485	2525	25	-800	-540	43.6	3.2
RISE 281	Bustrup	OxA-X-2627-26	3107	30	-1440	-1280	42	3.5
RISE 282	Hverrehus	OxA-30486	3148	27	-1500	-1310	42.4	3.3
RISE 326	Nybøl	OxA-32072	2995	27	-1380	-1120		3.4
RISE 432	Gjerrild	OxA-32087	3843	30	-2460	-2200	43	3.4
RISE 432	Gjerrild	OxA-32088	3906	28	-2480	-2290	42.2	3.3
RISE 433	Horne	OxA-32089	3362	28	-1750	-1560	42.3	3.3
RISE 460	Øster Herup	OxA-32093	3221	27	-1610	-1420	41.7	3.3
RISE 1280	Gjerrild	UBA-36752	4007	36	-2620	-2460		3.27
RISE 1281	Gjerrild	UBA-36753	3790	34	-2350	-2060		3.22
RISE 1283	Gjerrild	UBA-36754	3950	31	-2570	-2340		3.19

*AD

### Anthropology

Anthropological analyses were performed on all 88 individuals studied herein. Age and sex were determined from morphological features on the skull, pelvis, and teeth using standard methods outlined in [41–44]. In the cases where we were able to estimate age, the main categories of subadults and adults have additional subcategories (Table 2 and S1 Table). Subadults were divided into four age categories: young child (1–5 years), older child (6–11 years), juvenile (12–17 years) and subadult (<18 years). Furthermore, the group of adult individuals was divided into five age categories: young adult (18–25 years), middle adult (25–35 years), mature adult (35–45 years) and old adult (45+ years) or simply determined adult (>18 years). In cases where aging overlapped two categories, the adult age categories were pooled. The male and female categories were also pooled when analyzing the frequency of pathology (S2 Table).

**Table 2:** Strontium isotope, 14C results and sex and age determinations from individuals from the 3rd and 2nd millennia BC from Denmark presented in chronological order.

RISE#	Sitename	Region	Burial type	Period	^14^C BP	^87^Sr/^86^Sr	2s (abs)	Sex	Age	Age-group
RISE 273	**Gundsømagle mose**	Zealand	bog find	MN	**4578**	**0.709981**	0.000006	F	20–35	Young-Middle adult
RISE 65	**Kyndeløse**	Zealand	passage grave	MN	**4189**	**0.709984**	0.000013	F	20–30	Young-Middle adult
RISE 62	**Kyndeløse**	Zealand	passage grave	MN	**4187**	**0.710097**	0.000009	F	25–35	Middle adult
RISE 64	**Kyndeløse**	Zealand	passage grave	MN	**4145**	**0.710998**	0.000011	nd	25–35	Middle adult
RISE 61	**Kyndeløse**	Zealand	passage grave	MN	**4071**	**0.712588**	0.000011	M	20-(25)	Young adult
RISE 1280	**Gjerrild**	Jutland	Böstrup cist	MN	**4007**	**0.710026**	0.000006	nd	Inf	Subadult
RISE 66	**Kyndeløse**	Zealand	passage grave	MN	**3968**	**0.709904**	0.000012	M?	30–40	Middle-Mature adult
RISE 1283	**Gjerrild**	Jutland	Böstrup cist	MN	**3950**	**0.712764**	0.000009	F	21–30	Young-Middle adult
RISE 432	**Gjerrild**	Jutland	Böstrup cist	MN	**3906**	**0.710851**	0.000006	M	35–50	Mature-Old adult
RISE 73 a	**Gjrerrild**	Jutland	Böstrup cist	MN	**cd**	**0.710534**	0.000011	M	25–35	Middle adult
RISE 54	**Hellested**	Zealand	flat grave	LN I	**3796**	**0.711127**	0.000009	M?	21–23	Young adult
RISE 1281	**Gjerrild**	Jutland	Böstrup cist	LN	**3790**	**0.710018**	0.000008	nd	Inf	Subadult	
RISE 27	**Juelsberg**	Fyn	gallery grave	LN I	**3787**	**0.710141**	0.000012	M?	35–45	Mature adult
RISE 275	**Salpetermosen**	Zealand	bog find	LN I	**3751**	**0.710483**	0.000008	M	25–35	Middle adult
RISE 33	**Juelsberg**	Fyn	gallery grave	LN I	**3731**	**0.710142**	0.000011	M?	20–30	Young-Middle adult
RISE 39	**Marbjerg**	Zealand	gallery grave	LN I	**3727**	**0.710094**	0.000017	F?	35–45	Mature adult
RISE 34	**Marbjerg**	Zealand	gallery grave	LN	**cd**	**0.709371**	0.000010	F	25–40	Middle-Mature adult
RISE 35	**Marbjerg**	Zealand	gallery grave	LN	**cd**	**0.709896**	0.000014	M?	20–25	Young adult
RISE 105	**Dommergården**	Jutland	gallery grave	LN	**cd**	**0.710320**	0.000009	M?	30–35	Middle adult
RISE 63	**Kyndeløse**	Zealand	passage grave	LN I	**3721**	**0.709739**	0.000011	F	20–25	Young adult
RISE 25	**Juelsberg**	Fyn	gallery grave	LN I	**3705**	**0.709612**	0.000011	nd	25–35	Middle adult
RISE 53	**Hellested**	Zealand	flat grave	LN I	**3705**	**0.70959**	0.000012	F?	40–45	Mature adult
RISE 71	**Falshøj**	Jutland	megalithic tomb	LN I	**3701**	**0.711084**	0.000013	F	25–35	Middle adult
RISE 55	**Hellested**	Zealand	flat grave	LN I	**3700**	**0.711061**	0.000011	M	20–25	Young adult
RISE 57	**Hellested**	Zealand	flat grave	LN I	**3697**	**0.711054**	0.000013	M	*c*.18	Young adult
RISE 56	**Hellested**	Zealand	flat grave	LN I	**3689**	**0.710905**	0.000011	M	*c*.20	Young adult
RISE 42	**Marbjerg**	Zealand	gallery grave	LN I	**3681**	**0.709635**	0.000011	M	40+	Mature-Old adult
RISE 29	**Juelsberg**	Fyn	gallery grave	LN I	**3653**	**0.709804**	0.000009	M	35–50	Mature-Old adult
RISE 32	**Juelsberg**	Fyn	gallery grave	LN II	**3638**	**0.712103**	0.000009	F	25–35	Middle adult
RISE 30	**Juelsberg**	Fyn	gallery grave	LN I-II	**3627**	**0.711252**	0.000016	M	40+	Mature-Old adult
RISE 28	**Juelsberg**	Fyn	gallery grave	LN I-II	**3626**	**0.709705**	0.000013	nd	*c*.14-15	Juvenile
RISE 59	**Tummelhøj**	Jutland	gallery grave	LN I-II	**3618**	**0.711670**	0.000011	nd	Adult35-45	Mature adult
RISE 68	**Sejerslev**	Jutland	gallery grave	LN I-II	**3617**	**0.709280**	0.000013	nd	18–25	Young adult
RISE 69	**Falshøj**	Jutland	megalithic tomb	LN I-II	**3607**	**0.710522**	0.000013	F	30–35	Middle adult
RISE 14	**Langtved Faergekro**	Zealand	gallery grave	LN II	**3550**	**0.709991**	0.000008	nd	*c*.6	Older child
RISE 43	**Marbjerg**	Zealand	gallery grave	LN I-II	**3550**	**0.710038**	0.000011	M	40+	Mature-Old adult
RISE 31	**Juelsberg**	Fyn	gallery grave	LN II	**3542**	**0.710597**	0.000007	F	20–30	Young-Middle adult
RISE 45	**Gerdrup**	Zealand	gallery grave	LN II	**3539**	**0.711378**	0.000014	nd	12–16	Juvenile
RISE 67	**Sejerslev**	Jutland	gallery grave	LN I	**3535**	**0.709462**	0.000015	nd	20–35	Young-Middle adult
RISE 15	**Langtved Faergekro**	Zealand	gallery grave	LN II	**3527**	**0.709243**	0.000010	M	20–30	Young-Middle adult
RISE 44	**Marbjerg**	Zealand	gallery grave	LN II	**3522**	**0.709883**	0.000011	M	45+	Old adult
RISE 70	**Falshøj**	Jutland	megalithic tomb	LN II	**3518**	**0.710785**	0.000009	M	25–35	Middle adult
RISE 38	**Marbjerg**	Zealand	gallery grave	LN II	**3515**	**0.709714**	0.000013	M	35–45	Mature adult
RISE 40	**Marbjerg**	Zealand	gallery grave	LN II	**3504**	**0.711709**	0.000008	M	30–40	Middle-Mature adult
RISE 58	**Tummelhøj**	Jutland	gallery grave	LN II	**3502**	**0.710885**	0.000014	nd	20–30	Young-Middle adult
RISE 46	**Gerdrup**	Zealand	gallery grave	LN II	**3499**	**0.710324**	0.000012	nd	Adult	Adult
RISE 60	**Kyndeløse**	Zealand	passage grave	LN II	**3496**	**0.710211**	0.000012	F	30–40	Middle-Mature adult
RISE 36	**Marbjerg**	Zealand	gallery grave	LN II	**3493**	**0.709749**	0.000013	M?	20–25	Young adult
RISE 17	**Langtved Faergekro**	Zealand	gallery grave	LN II	**3488**	**0.710079**	0.000012	nd	*c*. 4	Young child
RISE 49	**Sebber skole**	Jutland	flat grave	LN II	**3452**	**0.710276**	0.000007	M	25–35	Middle adult
RISE 16	**Langtved Faergekro**	Zealand	gallery grave	LN II-EBA I	**3442**	**0.709672**	0.000007	M	25–40	Middle-Mature adult
RISE 37	**Marbjerg**	Zealand	gallery grave	LN II/ EBA I	**3429**	**0.709714**	0.000014	F	20–30+	Young-Middle adult
RISE 72	**Gjerrild**	Jutland	Böstrup cist	EBA I	**3410**	**0.709665**	0.000009	**nd**	Adult	Adult
RISE 41	**Marbjerg**	Zealand	gallery grave	LN II	**3405**	**0.709935**	0.000012	F?	30–40	Middle-Mature adult
RISE 26	**Kolby Kås**	Samsø	barrow	LN II—EBA I	**3362**	**0.710687**	0.000012	M	35+	Mature-Old adult
RISE 433	**Kimesbjerggårde**	Fyn	barrow	EBA I	**3362**	**0.710080**	0.000010	M	45–60	Old adult
RISE 51	**Sebber skole**	Jutland	flat grave	EBA I	**3360**	**0.710952**	0.000009	nd	18–25	Young adult
RISE 52	**Sebber skole**	Jutland	flat grave	EBA I	**3342**	**0.71096**	0.000013	M	20–30	Young-Middle adult
RISE 18	**Kolby Kås**	Samsø	barrow	EBA I	**3309**	**0.710555**	0.000010	nd	8–9	Older child
RISE 166	**Saltø**	Zealand	barrow	EBA I	**3304**	**0.711487**	0.000016	M?	25–35	Middle-Mature adult
RISE 169	**Store Havelse Strand**	Zealand	flat grave	EBA I	**3301**	**0.710840**	0.000006	M	25–35	Middle adult
RISE 104	**Jestrup**	Jutland	barrow	EBA III	**3295**	**0.71177**	0.000018	M?	20–30	Adult
RISE 50	**Sebber skole**	Jutland	flat grave	EBA I	**3274**	**0.710726**	0.000010	nd	Adult	Adult
RISE 460	**Øster Herup**	Jutland	barrow	EBA II	**3221**	**0.71401**	0.000001	nd	30–35	Middle adult
RISE 167	**Saltø**	Zealand	barrow	EBA I	**3220**	**0.712878**	0.000012	M?	40+	Mature-Old adult
RISE 168	**Saltø**	Zealand	barrow	EBA I	**3210**	**0.710128**	0.000012	nd	30–40	Middle-Mature adult
RISE 76	**Debel, Fur**	Jutland	barrow	EBA II	**3205**	**0.709370**	0.000013	M?	Adult	Adult
RISE 19	**Karlstrup**	Zealand	barrow	EBA II	**3196**	**0.71140**	0.000015	nd	7–8	Young child
RISE 20	**Karlstrup**	Zealand	barrow	EBA II	**3162**	**0.717881**	0.000011	M	25–40	Middle-Mature adult
RISE 48	**Sebber skole**	Jutland	flat grave	EBA II	**3156**	**0.710075**	0.000009	M?	40–50	Mature-Old adult
RISE 47	**Sebber skole**	Jutland	flat grave	EBA II	**3153**	**0.710206**	0.000016	M	25–35	Middle adult
RISE 282	**Hverrehus**	Jutland	flat grave	EBA II	**3148**	**0.710205**	0.000012	nd	*c*.5	Young child
RISE 23	**Debel**	Jutland	barrow	EBA II	**3117**	**0.708713**	0.000017	M	20–25	Young adult
RISE 281	**Bustrup**	Jutland	barrow	EBA II	**3107**	**0.711165**	0.000012	nd	*c*.15	Juvenile
RISE 21	**Karlstrup**	Zealand	barrow	EBA II	**3092**	**0.716564**	0.000009	M	20–25	Young adult
RISE 22	**Gjessinggård**	Jutland	flat grave	EBA II-III	**3053**	**0.709881**	0.000006	F	30–40	Middle-Mature adult
RISE 326	**Nybøl**	Jutland	barrow	EBA III	**2995**	**0.711714**	0.000011	M	25–35	Middle adult
RISE 106	**Nørhågård**	Jutland	barrow	EBA III	**2949**	**0.7104631**	0.000011	M?	25–35	Middle adult
RISE 13	**Strandfogedgård**	Zealand	barrow	EBA	**cd**	**0.7107223**	0.000007	nd	*c*.8	Older child
RISE 78	**Strandfogedgård**	Zealand	barrow	EBA	**cd**	**0.710140**	0.000013	nd	Adult	Adult
RISE 79	**Strandfogedgård**	Zealand	barrow	EBA	**cd**	**0.714440**	0.000014	nd	25–35	Middle adult
RISE 107	**Sennels**	Jutland	barrow	EBA	**cd**	**0.708972**	0.000012	nd	Inf	Subadult
RISE 108	**Vorupørvej 16**	Jutland	barrow	EBA III	**cd**	**0.710169**	0.000011	nd	13–15	Juvenile
RISE 170	**Ballehøj**	Fyn	barrow	EBA II	**cd**	**0.710101**	0.000007	M?	20–25	Young adult
RISE 24	**Ballermosen**	Zealand	flat grave	BA?	**na**	**0.710649**	0.000014	M	50+	Old adult
RISE 276	**Trundholm mose**	Zealand	bog find	LBA V/VI	**2525**	**0.710053**	0.000010	M	40–60	Old adult
RISE 274	**Egedal mose**	Zealand	bog find	EIA	**2068**	**0.711437**	0.000009	M	35–45	Mature-Old adult
RISE 12	**Sønderhå**	Jutland	stone cist	EIA	**1949**	**0.710168**	0.000005	F	18–20	Young adult

^cd^ contextually dated

MN = Middle Neolithic; LN = Late Neolithic; EBA = Early Bronze Age; BA = Bronze Age; LBA = Late Bronze Age; EIA = Early Iron Age

Skeletal and dental pathologies, signs of trauma or anomalies were noted (S1 Table and S2 Table) for all 88 individuals studied herein. The different levels of preservation limited the observations significantly.

Previous results of stature estimation were available for 7 individuals (S1 Table). These were either based on the length in the grave or calculated based on the length of a long bone (femur, tibia or humerus) using the method of [45]. More recent studies on stature among prehistoric individuals [32] have utilized the method by [46], as it is developed on European Holocene individuals. It was possible to retake femur length measurements of three individuals (S1 Table), but a stature estimation using [46] was not attempted as it could not be compared to the previously reported stature estimates of the Danish Bronze age individuals herein.

## Results and discussion

In recent years, a number of human mobility studies based on strontium isotope analyses of human remains from among others, southern Sweden, Germany and Britain have revealed indications of a rather high rate of human mobility during the 3^rd^ and 2^nd^ millennia BC e.g. [47, 48–52].

Some of these studies suggested a pattern in which exogamy may have prevailed during the Corded Ware and Bell Beaker/Early Bronze Age societies, as a majority of the women investigated were of non-local origin [3, 50]. In other cases, like in the multi-isotopic investigations of skeletal remains of 264 individuals from the British Chalcolithic–Early Bronze Age, results indicated a high degree of mobility but with “little difference between male and female migration histories across Britain” [51].

Another study based on investigations of the human remains (mostly of young males) excavated from the Bronze Age battlefield of Tollense (c. 1250 BC) in northern Germany, revealed that a large majority of these individuals were of non-local origin, and that they potentially originated from various places characterized by different geologies [47]. A somewhat similar case to the Tollense study may be found in the unusual Late Bronze Age cemetery of Neckarsulm in south-western Germany where only males were buried. The strontium isotope analyses conducted on individuals from this site revealed that one third of these individuals were also of non-local origin [53].

Yet another recent study from southern Sweden, based on multi-isotopic analyses of 61 individuals dating from 2300–1200 BC, suggested that mobility was rather high in this region too, but no differences with respect to social status or sex of individuals investigated could be seen [49].

Taken together, these studies reveal a highly complex Bronze Age society.

With respect to ancient DNA characterization, only a few individuals from present-day Denmark from this period have been analyzed thus far [1]. Our strontium isotope analyses encompass five of these individuals, and their overall genomic information resembles the typical Corded Ware-like gene pool, typical for northern and central Europe at this time [1]. More specifically, they all have the genomic "steppe signature" that ultimately derives from the Yamnaya-culture-related people who expanded into central and northern Europe shortly after 3000 BC [1, 2]. The Corded Ware and related cultures somehow emerged from this admixture between Yamnaya-related and the Late Neolithic population and started expanding across large parts of Central and Northern Europe. This seems to imply that the population we investigate in this study might represent a newly genetically transformed population.

The results of our radiocarbon analyses are presented in Table 1. The calibrated age ranges in the table as well as in the text correspond to 95.4% probabilities [40]. Our results reveal that one individual dates to the 4^th^ millennium BC and three individuals yielded radiocarbon ages younger than the 2^nd^ millennium BC. The remaining individuals yielded radiocarbon ages ranging from the 3^rd^ to the 2^nd^ millennia BC.

The results of our strontium isotope analyses are presented in Table 2 and listed in chronological order according to the radiocarbon dates (in sites with multiple individuals we start with the oldest radiocarbon individual). The strontium isotope data set reveals a wide range of values from ^87^Sr/^86^Sr = 0.70871 (RISE 23, from the site of Debel) to ^87^Sr/^86^Sr = 0.71788 (RISE 20, from the site of Karlstrup). Despite the difficulties of establishing the baseline range some of the herein investigated individuals may be classified as non-locals. A few individuals have tooth enamel signatures that lie just above the upper baseline limit of ^87^Sr/^86^Sr = 0.711 and therefore, the classification of these humans as non-locals should be considered with caution. Nevertheless, the significant proportion of individuals with relatively radiogenic values suggest that about a quarter of the individuals studied herein seem to have originated from other places than from those they were buried, and hence implying a continuous degree of mobility during the 3^rd^ and 2^nd^ millennia BC.

Our anthropological investigations reveal that out of the 88 individuals investigated, 75 were adults: 45 were males (including 15 possible males), 17 were females (including 3 possible females) and 13 were adults of undetermined sex (Table 2 and S1 Table). Furthermore, we could identify 13 subadults. The state of preservation limited the investigation of pathological alterations on the skeletons as well as the possibility of diagnoses (S1 Table and S2 Table).

### 3^rd^ millennium BC (Middle Neolithic to Late Neolithic I, 3300 BC—2000 BC)

#### Middle Neolithic

The earliest dated individual from our dataset, a bog find of an adult female, aged 20–35 years (RISE 273) from Gundsømagle Mose, Zealand (Fig 1 and S1 File) yielded a radiocarbon date of 3500–3110 cal BC (Early Neolithic II- Middle Neolithic A). Our strontium isotope analysis yielded ^87^Sr/^86^Sr = 0.7099, compatible with a local provenance. Her skull displays evidence of an unhealed blunt force trauma to the back of the head. The unhealed state suggests that a blunt instrument or surface struck her head near or at the time of death and that the ensuing trauma may have caused her death. Cranial trauma is a common feature in human bog finds from the Early and Middle Neolithic [54, 55].

The double passage grave of Kyndeløse (Fig 1, S1 File) located on the island of Zealand yielded 70 individuals as well as a large number of grave goods, including flint artefacts, ceramics, and tooth and amber beads. We conducted strontium isotope analyses of seven individuals from Kyndeløse encompassing a period of c. 1000 years, indicating the prolonged use of this passage grave. The oldest of the seven individuals is a female (RISE 65) from whom we measured a “local” strontium isotope signature (^87^Sr/^86^Sr = 0.7099). Similar values were measured in five other individuals, including adult males and females. Only a single individual from Kyndeløse, an adult male (RISE 61) yielded a somewhat different strontium isotope signature of ^87^Sr/^86^Sr = 0.7126 which seems to indicate a non-local provenance. The skull of this male individual revealed healed porosities in the eye orbits, *cribra orbitalia*, a condition which is possibly linked to a vitamin deficiency during childhood, such as iron deficiency.

**Fig 2:**
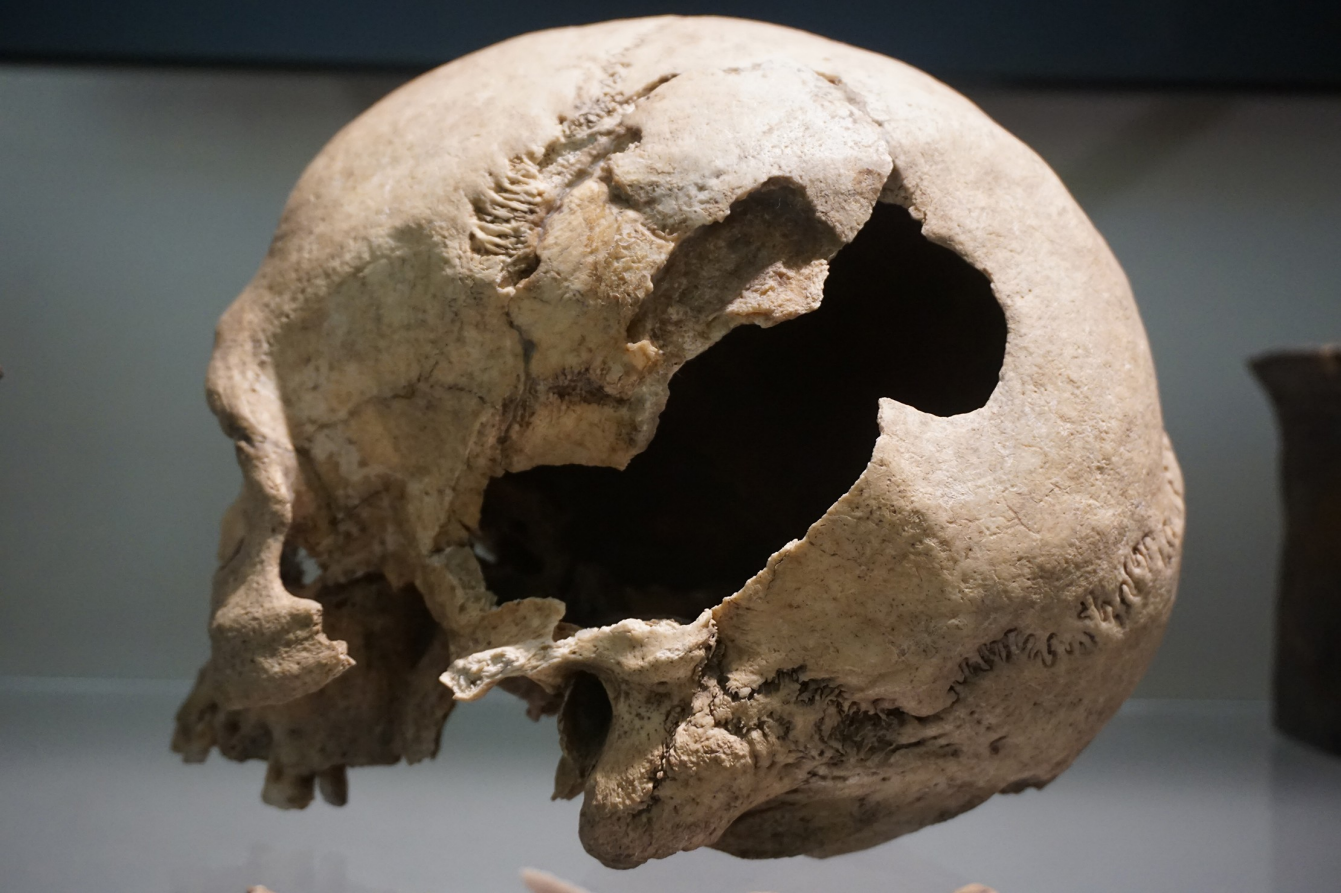
Cranium from individual RISE432 excavated from the Gjerrild burial site that shows a healed trepanation. (Photo: Samantha S. Reiter, National Museum of Denmark).

**Fig 3:**
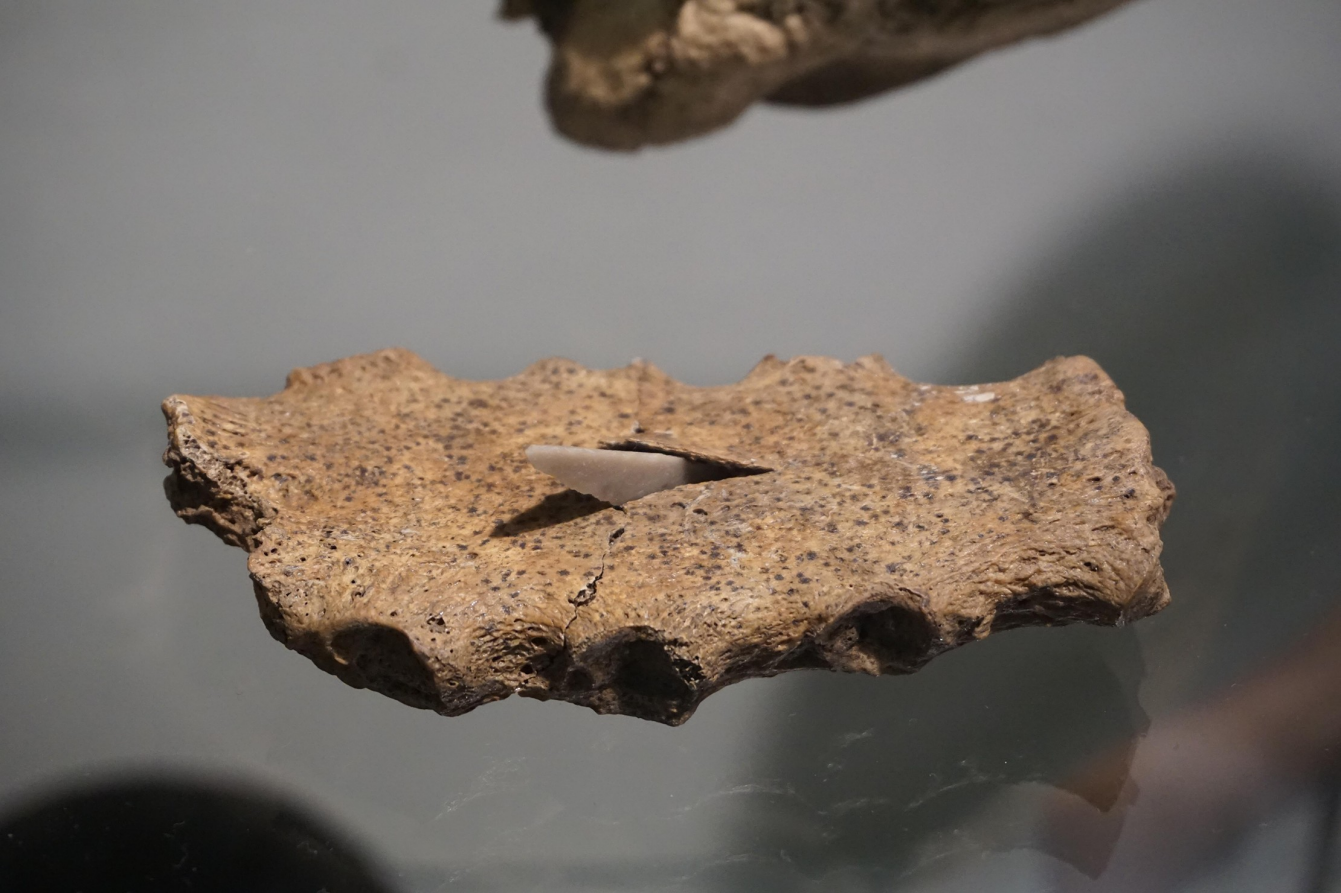
Arrowhead in the sternum of individual RISE 73a, 1282 excavated from the Gjerrild burial site. (Photo: Samantha S. Reiter, National Museum of Denmark).

From the Single Grave Culture (SGC) which is closely related to the Corded Ware Complex in central and eastern Europe and dates from c. 2800 BC to 2200 BC, we analyzed seven of the at least ten individuals who were buried at the site of Gjerrild in eastern Jutland (Fig 1). Gjerrild is a key SGC site, as to date it has provided the most substantial skeletal material pertaining to this culture from present-day Denmark. However, it is not a typical SGC grave, but a megalithic chamber of the so-called “Bøstrup type”. The SGC pottery was decorated with cord or stamp impressions and the stone battle axes were a common feature of male equipment. Such shared traits in the Corded Ware Complex probably reflected shared occupational, social and religious characteristics. Apart from one individual who yielded a Bronze Age date, five individuals date within the period that spans from c. 2600 BC to 2200 BC, hence representing the middle and late SGC phases (Table 1 and S1 File). Of the seven individuals, three males, one female, two infants and one adult (only represented by a disarticulated mandible, and dated to the Bronze Age), all but one yielded strontium isotope signatures that fall within the local baseline range. Only the female (RISE 1283) has a more radiogenic strontium isotope signature of ^87^Sr/^86^Sr = 0.7127, which is similar to that of the male from Kyndeløse and might indicate non-local provenance. One of the individuals at Gjerrild, a mature-old adult male, who yielded a local signature (RISE 432) was accompanied by a D-type arrowhead and an amber bead which lay on his right side. He showed signs of inflammation on his lower legs, in particular on the left one. He had a healed trepanation (Fig 2). Another individual (RISE 73a, 1282), an adult male, was found with a type D arrowhead in the sternum (Fig 3).

#### Late Neolithic I

We sampled individuals from a total of twelve different sites that date to the Late Neolithic period (2300/2250-1700 BC). One of these sites is Hellested on Zealand (Fig 1 and S1 File), with four flat graves containing five individuals, four young males and one mature adult female. We conducted strontium isotope analyses of enamel from all five individuals, and our results point to two individuals being characterized by local strontium isotope values. One of these individuals, the female, was buried with no grave goods (RISE 53, grave B) while the other, a young male, was buried with a fragmented bone pin (RISE 56, grave F). The other three male individuals (RISE 54, 55, 57) yielded similar strontium isotopic values that lie slightly above the local baseline range. All these individuals had been buried with early flint daggers (type I and II), and one of them (RISE 57, grave A) additionally had a ring-headed pin (Ringkopfnadel) [56]. On the basis of the presence of this ring-headed pin, Lomborg [56] suggested that these individuals had connections with the Únětice culture. Furthermore, three of them have radiocarbon dates that overlap (RISE 55, 56 and 57; Table 1).

Another Late Neolithic site is Juelsberg on the island of Funen (central Denmark, Fig 1 and S1 File) which is a gallery grave that contained at least 19 individuals. We conducted strontium isotope analyses of tooth enamel on 8 out of the 19 individuals and two of them, a male and female, yielded ratios that suggest a non-local origin (RISE 30 and 32). The grave goods comprise a (Lomborg) type I flint dagger but also some non-local type of artefacts. These consist of an early type of bone pin (type 7) mainly found in south-eastern Scandinavia, and a barbed and tanged flint arrowhead of the west-European Bell Beaker type suggesting western connections. The middle adult female (RISE 32) yielded an ^87^Sr/^86^Sr = 0.7121 and the mature to old adult male (RISE 30) yielded a ^87^Sr/^86^Sr = 0.7112. The different Sr isotope signatures of these individuals imply that they might have originated from different areas, albeit their radiocarbon dates are very similar.

The gallery grave of Marbjerg, Zealand (Fig 1), yielded 17 individuals (S1 File), and we conducted strontium isotope analyses of tooth enamel on 11 of them. The majority of the individuals were males, but females and children, too, were present. Anthropological investigations of the individuals from this site, males as well as females, indicate a relatively high life expectancy with respect to that typical for this period (S1 Table). Our radiocarbon dates revealed that this grave was in use for several hundred years from the Early Late Neolithic (2210–2030 cal BC, RISE 39) to the Late Neolithic /Early Nordic Bronze Age Period (1770–1620 cal BC, RISE 41). Despite the long-term use of this grave, 10 of the 11 individuals studied herein yielded a very narrow and overlapping range of strontium isotope values between ^87^Sr/^86^Sr = 0.7096–0.7101. Their values suggest not only that these individuals were local but that their food sources were derived from the same area over the course of several centuries. Only the tooth enamel sample of one individual, a middle to mature adult male (RISE 40), yielded a higher value of ^87^Sr/^86^Sr = 0.7117, which seems to suggest a non-local origin.

### 2^nd^ millennium BC (Late Neolithic (II) to Early Bronze Age (I-III) (2000–1100 BC)

#### Late Neolithic II

From the gallery graves at Sejerslev and Tummelhøj (Fig 1), both located on northern Jutland, we investigated four individuals dating to the Late Neolithic II. One of these individuals from the site of Tummelhøj (RISE 59) yielded a Sr isotopic composition that is different from the baseline (Table 2). This individual, a mature adult, show signs of ante-mortem tooth loss where the *alveolar* had begun to heal with some inflammatory response (S1 Table and S2 Table). Six other individuals from two gallery graves on the island of Zealand, Gerdrup and Langtved Færgekro, dating to the Late Neolithic II, were also investigated. Five of these six individuals yielded Sr isotope values that point to local provenance.

From the megalithic tomb of Falshøj situated in Jutland, we investigated three individuals, one of whom was a female, dating to the Late Neolithic (RISE 71). Here, two of the individuals seem to be of local provenance (RISE 69 and 70).

#### Early Bronze Age

While most of the elite of the Early Nordic Bronze Age were buried within burial mounds, often in some kind of stone and/or wooden coffin e.g. [7, 57], the flat graves of this period appear to represent the non-elite, i.e., the commoners [49]. While it is the commoners who represent the largest part of the society, yet they are typically much less visible in the archaeological record than the elite [49]. In present-day northern Denmark, seven flat graves containing at least ten individuals were unearthed at the site of Sebber Skole located in northern Jutland (Fig 1). Only one of the burials contained grave goods consisting of a (Lomborg) type VI flint dagger dating to Period IB (1600 BC -1500 BC) of the Early Bronze Age [58]. This date was confirmed by our radiocarbon dates which demonstrated, moreover, that this cemetery was in use from the Late Neolithic to the Early Bronze Age (Period II) (Table 1). The burial in a shell-midden without any grave goods except for a flint dagger [58] suggests that these individuals represent the non-elite. We conducted strontium isotope analyses of tooth enamel from six individuals, all of whom yielded strontium isotope values between ^87^Sr/^86^Sr = 0.710 to 0.711 that fall within the local baseline.

Results from individuals from the remaining Bronze Age flat graves studied herein, i.e. Hverrehus and Gjessinggård from Jutland, and Store Havelse Strand and Ballermosen from the island of Zealand (Fig 1), are also compatible with the baseline range of present-day Denmark.

**Fig 4:**
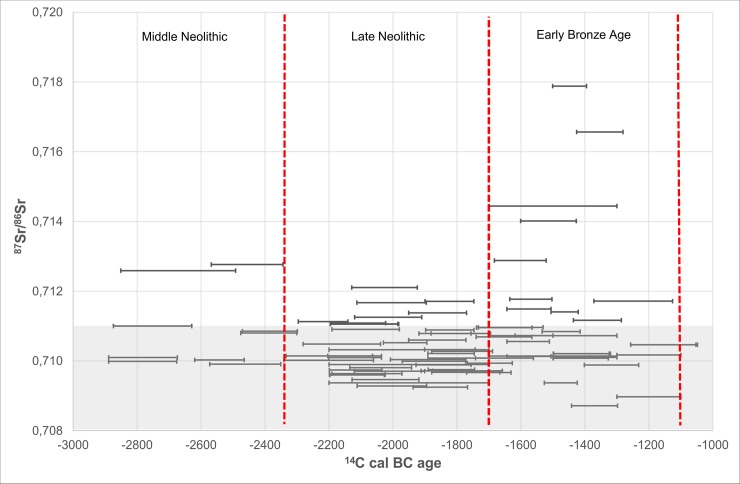
Diagram plotting results of strontium isotope ratios versus calibrated radiocarbon dates of the individuals investigated. The grey band shows the “local” baseline (for details see baseline section).

From around 1600 BC, a change appears to occur with respect to mobility as seen in Fig 4. From this point in time our dataset shows that some individuals have Sr isotope values above 0.713, i.e. values that do not seem to be represented by any of the individuals studied herein from the previous periods. Individual (RISE 460) yielded an enamel ^87^Sr/^86^Sr = 0.714 which represents the earliest strontium isotope ratio above 0.713 in our dataset. This individual, an adult of c. 30 to 35 years of age, was unearthed from a stone cist inside the burial mound of Øster Herup in Jutland. The radiocarbon analyses revealed an age of 1610–1420 cal BC (Early Bronze Age, Period I-II).

The burial mound of Karlstrup near Copenhagen (Fig 1 and S1 File), also yielded individuals with Sr isotope values above 0.713 (Table 2). The mound contained four gallery graves and a number of inhumations representing at least 31 individuals [59] (S1 File). We analyzed material from the central grave containing the remains of three individuals, the skeletons of two adult males (RISE 20 and 21) and the mandible of a child (RISE 19). The two male individuals were buried with a large number of bronze objects in Nordic style as well as other material (S1 File) [59]. The grave goods indicate that they belonged to the elite, and based on the objects, it may be assumed that they were locals. The radiocarbon dates revealed that all three individuals lived during the Nordic Bronze Age period II (1500 BC- 1300 BC, Table 1). The two male individuals share some physical features, one of them being furrows that indicate the use of toothpicks. Living stature was not calculated, but the femoral lengths (50.5 cm for RISE 20 and 47.5 cm for RISE 21) indicate that these individuals would have been quite tall while alive compared to other prehistoric populations in Denmark and Europe [32, 46]. Previous studies suggest that body height and body size of humans increased during the Late Neolithic and Early Bronze Age in Denmark [60]. These changes in body features have been interpreted as being caused by human migration from central Europe [1, 61]. The two adults have the highest strontium isotope ratios in our dataset (RISE 20, ^87^Sr/^86^Sr = 0.7178, and RISE 21, ^87^Sr/^86^Sr = 0.7165), indicating an old geological terrain as their homelands. Within the Nordic region, such high strontium isotopic values can be found in areas of, e.g. Sweden, Norway, and on the island of Bornholm [35, 62–65]. However, a few areas characterized by such high bioavailable Sr isotope signatures can also be found in central Europe and the British Isles, e.g. [26, 66–68]. Regardless of the difficulties of determining their provenance, our results suggest a highly dynamic and complex socio-economic pattern.

We also analyzed one of the well-known oak-coffin burials from the Nordic Bronze Age, the Nybøl male, unearthed in southeastern Jutland (Fig 1). The textiles with which the Nybøl male was buried have been investigated earlier by several scholars using different methodologies [13, 69]. The strontium isotope analyses of wool samples from the textiles of the Nybøl male indicated a wide range of strontium isotope values (^87^Sr/^86^Sr ∼0.715–0.725), suggesting that the textiles were made of wool coming from a variety of areas [13]. Our strontium isotope analyses of the skeletal remains of the Nybøl male (RISE 326), who is estimated to have been between 25 to 35 years of age when he died, suggests that he might also have been of non-local origin. The artefacts in the Nybøl burial are however of Nordic type: a razor and a comb [70]. His oak coffin has been dendrochronological dated to between 1277–1246 BC previously [71].

**Fig 5:**
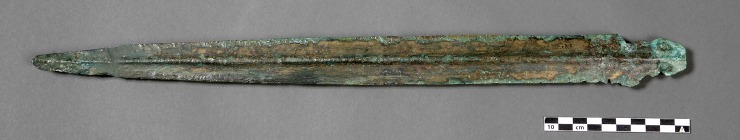
Image of the bronze sword of the “Rixheim” type from the Jestrup male grave. (Photo: Klaus Madsen, courtesy of Museum Thy).

Another elite individual who yielded a Sr isotope value which suggests non-local provenance is the adult male warrior from Jestrup (RISE 104) who was buried in a stone cist inside a burial mound in the Thy area (Fig 1) [72, 73]. This region was densely populated and central in terms of the economy during the Early Bronze Age [15]. His grave goods consist, among others, of a sword of the Rixheim type (Fig 5) which was commonly distributed in south-west Europe (south-east France/Switzerland), suggesting a potential origin south of present-day Denmark (S1 File). However, the fibula and a bronze double button are of Nordic type. This grave represents one of ten graves in the Thy area containing a pan-European assemblage of grave goods from this period (Late Bronze Age period II; 1500 BC -1300 BC). They have a common assemblage of grave goods which is less diverse compared to that of the other burials which contained local swords [74].

### Comparing 3^rd^ and 2^nd^ millennia mobility

Our results indicate a change from around 1600 BC onwards, as individuals with Sr isotopic values above 0.713 start to appear in our dataset and suggest mobility. Furthermore, the large range of values (between ^87^Sr/^86^Sr = 0.713 to 0.718) represented by these individuals imply that the areas which the non-locals individuals migrated from were geographically diverse and might have included more distant regions.

The shift in human mobility characterized by the expansion in diversity of areas of origin of the non-locals appears to occur parallel to the emergence of the long-distance metal trade that connected present-day Denmark to areas in, e.g., central and southern Europe as well as the British Isles e.g. [4, 5, 12]. Moreover, it appears that mobility is most evident within the group of individuals buried in barrows, compared to those in flat graves (all the nine Early Bronze Age flat graves herein investigated suggest “local” origin) (Table 2). This differs from the recent results from Scania in southern Sweden which do not seem to show differences with regards to mobility and social status [49]. This aspect attests to potential differences within the Nordic Bronze Age region.

**Fig 6:**
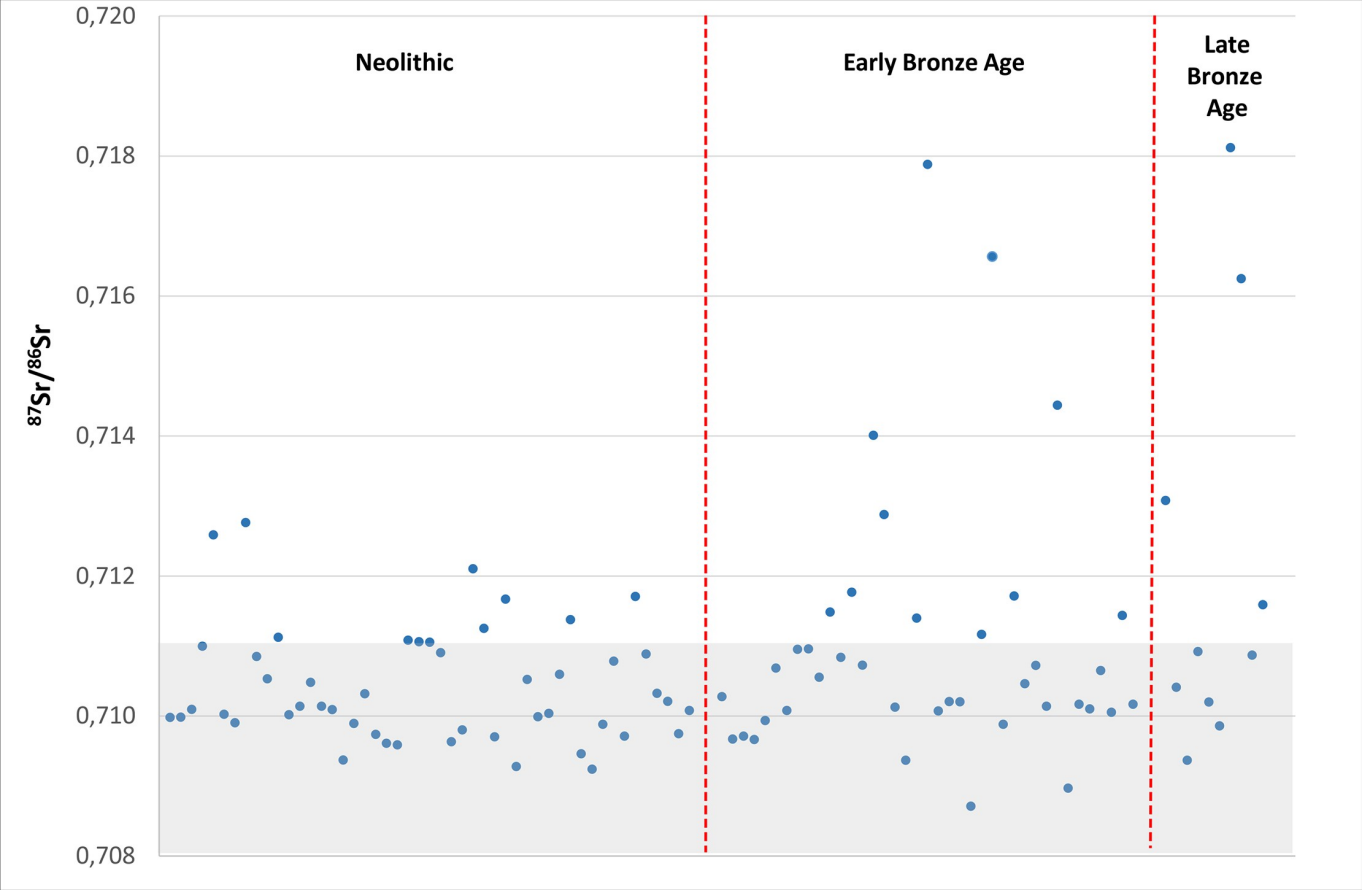
Strontium isotope results of the 88 investigated individuals including Late Bronze Age individuals investigated previously. The grey band shows the “local” baseline (for details see baseline section).

A previous study appears to indicate that this mobility pattern, including the expansion trend with respect to the diversity of areas, continued into the Late Bronze Age (1100–500 BC) [18] as well (Fig 6). This pilot case study of strontium isotope analyses on cremated human remains was based on analyses performed on individuals excavated from Funen and Jutland. Three individuals revealed Sr isotopic values above 0.713 [18].

When comparing our study with other recent similar investigations within the 2^nd^ millennium in Europe, a quite complex picture emerges what seems to include different mobility patterns depending on the areas. While in southern Sweden tooth enamel strontium isotope analyses revealed that both males and females of varying socio-economic status and wealth migrated to the area during the Nordic Bronze Age [49], other investigations in, e.g. the Lech Valley area in southern Germany, point to a high degree female mobility [48]. Yet another recent study from Northern Italy also reports mobility mostly of women in which appears to have been a patrilocal society [37].

In sum, our study provides new insights into mobility during a crucial point in time at the beginning of the Nordic Bronze Age. This mobility might have caused a rapid homogenization of gene pools. While it will be desirable and very relevant to discuss the genetic results more in detail with the herein presented strontium isotope results and their potential implications for Europe-wide population dynamics and mobility from particularly the “steppe” people, we consider that with only the few samples at hand (five) it would be too premature to expand on this issue at this stage. There is a clear and strong need for further aDNA analyses on more individuals to better understand the detailed levels of the macro-socio-dynamics during the 3^rd^ and 2^nd^ millennia in present-day Denmark. Nevertheless, our results provide new information suggesting the emergence of new and potentially long-distance alliances that seem to have been established during the Early Nordic Bronze Age. Even if the Bronze Age displayed strong regional cultural patterns, such as the Nordic Bronze Age culture and the Tumulus Culture [9], travel and migration between different regions and cultures were evidently substantial, and some newcomers appear to have been integrated into the local society. Finally, our results emphasize the need for further multidisciplinary and multi-analytical investigations when studying socio-dynamics in prehistory.

## Conclusions

We have conducted a multi-analytical investigation on the largest data-set to date, composed of 88 individuals excavated from 37 localities within present-day Denmark and dating to the 3^rd^ and 2^nd^ millennia BC in order to map human mobility. Our large study allowed us to observe the variations of mobility throughout this period. The strontium isotope results, combined with radiocarbon dating efforts, indicate a clear shift in migration patterns from around 1600 BC onwards, distinguished by mobility from a large variety of regions potentially with diversified and different geological backgrounds (and potentially more distant from present-day Denmark). This change in migration pattern appears to have occurred during a key period when the Nordic Bronze Age society flourished parallel to the emergence of the long-distance trade of metals and when society experienced a hitherto unseen economic growth suggesting that these aspects are closely related.

## Supporting information

S1 File Site description of the burials and their contexts. (PDF)

S1 Table Overview of the anthropological data. (PDF)

S2 Table Overview of the most common skeletal and dental pathologies. (PDF)
