# Supplementary material for: Mapping human mobility during the third and second millennia BC in present-day Denmark
Source: PLoS One. 2019 Aug 21;14(8):e0219850. doi: 10.1371/journal.pone.0219850 (PMC6703675; doi:10.1371/journal.pone.0219850)
Supplement: S1 File — (PDF) [file pone.0219850.s001.pdf]

S1 Appendix

Site descriptions

By Sophie Bergerbrant and Karl-Göran Sjögren

The appendix is structured according to the Danish system of county (*amt*), district (*herred*) and parish (*sogn*). Some of the sites have different names in reports, publications etc, or have changed their spelling after the discovery of the find. Here, in an effort to standardize and follow modern usage, the names are those found in *Die Funde der älteren Bronzeziet des nordischen kreises in Dänemark, Schleswig-Holstein und Niedersachsen: Fredriksborg und Københavns Amt.* Sb numbers (Sb=sogneberetning) denotes numbers in the Danish sites and monuments register, Fund og Fortidsminder (Cultural heritage database; http://www.kulturarv.dk/fundogfortidsminder/). PMD numbers refer to the book *Prehistoric Man in Denmark* (1956). Roman numerals for some skulls refers to numbering given in the National Museum of Denmark. For RISE numbers, laboratory numbers see Table 1 and Table 2 main text.

Ballermosen, Jægerspris, Dråby parish sb no 320, Horns District, Fredriksborg County

Barrow/flat grave

The remains of this grave were discovered by the military in 1958, having been exposed from a ploughed-out barrow or flat grave [1]. The grave contained traces of a wooden coffin, some human skeletal remains and possible indications that a small bronze object had been placed in the grave [1]. The sample from this individual, RISE 24, yielded no radiocarbon date, but the location of the grave and its shape and form indicate that it can be dated to the Early Bronze Age or possibly Late Neolithic. In the AS's (*Antropologiske Samling)* archive the site is called Jægerspris hovedgåard.

Salpetermosen, Hillerød parish Sb no 8, Lynge-Fredriksborg District, Fredriksborg County

Bog find

In 1947 human remains were found together with a bronze dagger (NM B 13793) in a bog in Hillerød parish (010301-8 [2]). The project has analysed the human remains (RISE 275). The dagger has been typologically dated to 900-500 cal BC [3] but according to radiocarbon dating the skeletal remains date to the Late Neolithic I (2290-2030 cal BC).

Store Havelse Strand (Maxivej), Ølsted parish sb no 78, Strø District Fredriksborg County

Flat grave cemetery

A flat grave cemetery was excavated 1986 by Steffen Stumann Hansen. The site contained six graves, five inhumations and one cremation [4]. Grave A, a Late Bronze Age urn contained cremated bones. It was placed in the area of the knees in grave B. The latter grave contained the remains of an adult female, though the skull is missing. It was partly placed over graves C and D [4]. Grave C contained skeletal human remains and a belt hook. This grave was dated to PII by the excavator [4]. Grave D contained the remains of a child placed in hocker position. Grave H contained human skeletal remains, two spiral bronze earrings and six dog teeth from at least three different dogs. The last burial (grave J) contained the possibly modern remains of a calf that was skinned but not eaten [4].

The project analysed grave C (RISE 169). It measured 181.5 cm in the grave [4]. The radiocarbon date places the grave in Period I (1640-1500), and based on the belt hook it is likely that the burial can be placed in Period IB, rather than Period II, which had previously been proposed.

Egedals mose, Ølstykke parish sb no 64, Ølstykke District, Fredriksborg County

Bog find

In 1944 a skeleton was found in this bog. Eight years later a bronze sword was found in the same area during peat digging (010607-64 [2]). The skeletal remains were sampled for this project (RISE 274) and the remains were identified as those of a male, 35-45 years old. The radiocarbon date showed that the human remains belonged to the Iron Age (180 - 1 cal BC), i.e. not contemporary with the sword (NM B 14367).

Gundsømagle Mose, Hove Å, Hvedstrup parish Sb no 29, Smørum District, København County

Bog find

Remains of several individuals were found in different areas of Hove Å between 1940 and 1945. One skeleton was found with part of a log-boat, and was determined to be a male with worn teeth, aged 18-21 [5]. This skeleton dates to Early Bronze Age, 1450-1010 cal BC (K-6301 3020 BP ±85 $\delta^{13}C$ -18.8, 020212-48 [2], [6]).

In the project, a skull (RISE 273) from a different individual was analysed. It was found together with an ornamented arrowhead that dates to the Early Iron Age (020406-29 [2]); the radiocarbon date of RISE 273 dates it to the Early Neolithic II - Early Middle Neolithic A (3500-3100 cal BC).

Marbjerg (Grydhøj), Fløng Parish Sb no 16, Sømme District, København County

Gallery grave

This gallery grave was excavated in 1911 by Hans Kjær [7, 8]. It was a small cist of Zealand type, c. 3.6 by 0.85 m, dug c. 0.9 m into the ground and covered by a low mound [7, 8].

The find material was sparse and consisted of a flint dagger of type VA [7, 9], two slate pendants of type A, one bone pin, one bone awl and four dog teeth. Outside the gallery grave was a small stone cairn which contained another slate pendant [7].

The gallery grave contained at least 17 individuals, of which eight were males, three were females and three were subadults [7, 10]. One skeleton, RISE 38 (PMD 96), was articulated, lying extended on its back with the head towards the west. It has been suggested that this individual was the last person to be buried at the site. This is contradicted by the radiocarbon date, according to which this individual should be in the middle of the burial sequence (Table 1 main text). The other individuals were all disarticulated [10].

In this project 11 individuals were analysed (RISE 34-44). Only one of the samples produced aDNA results (RISE 42) in a previous study [11] revealing a haplogroup H3v+16093 and genetic data determined it to be male. The radiocarbon dates range from the early stages of the Late Neolithic, 2210-2030 cal BC (RISE 39) to Bronze Age Period I, 1770-1620 cal BC (RISE 41).

Gerdrup, Kirkerup parish Sb no 20, Sømme District, København County

Gallery grave

David Liversage excavated this grave in 1963 [7, 12]. The tomb was a gallery grave of Zealand type, partly covered by a mound containing Bronze Age burials. The cist was dug ca 0.8 m into the ground and was c. 2.5 by 0.8 m large [7].

At least 16 individuals were buried in the grave, of which 11 were adults and 5 were subadults represented by minimum eight males and three females [13]. One skeleton was almost intact, and lay extended on his back with the head to the NW. Liversage suggested that this individual was the last person buried. A flint dagger of Lomborg type V was found by his right hand [12].

Inside the entrance, and on top of the feet and lower legs of the articulated skeleton, was a concentration of disarticulated human remains from several individuals. Stratigraphically below the articulated skeleton were remains of earlier burials. Apart from an articulated foot and a skull with mandible, these remains were all fragmentary and disarticulated. Scattered human bones were also found outside the entrance. In a few cases, these could be shown to belong to the same individuals as bones found in the chamber. Finally, bones of a child were found outside the SW chamber wall [12].

In front of the entrance, a young female had been buried in a flat grave, probably in a coffin. No artefacts were found in this grave [12]. Objects found in the cist included a dagger Lomborg type VB, a bone pin Ebbesen type 7, a bone awl, a bone bead, a bronze finger ring and fragments of two other bronze rings [7, 12].

Remains of two individuals were analysed: RISE 45 (an individual of c. 12-16 years of age) and RISE 46 (an adult individual, sex was not possible to identify). Details of their position are not available. Both date to the Late Neolithic II (1960-1770 and 1910-1740 cal BC).

Karlstrup, Karlslunde parish Sb no 4, Tune District, København County

Gallery graves and barrow

Christian Leif Vedbæk excavated the mound in Karlslunde parish in 1965-66. During the excavation four gallery graves and twelve inhumations were found. In total at least 31 individuals were buried in the mound [7, 14, 15]. The skeletal material was sent to the *Antropologiske Samling* before the final numbering of the graves were made. Therefore, the grave numbers in AS's catalogue and the report are not correlated. There is a key to the renumbering in their archive. The key and the archaeological report (text and photographs) were used to identify the skeletons that were analysed. The grave number for each grave are

also different in catalogue of Aner and Kersten [14] than in the archaeological report. In Aner and Kersten [14] all graves are numbered from A and onwards.

In this project the double grave XVI (Aner & Kersten grave Q) was analysed. It contained the remains of two individuals: Skeleton A (RISE 20) and Skeleton B (RISE 21). Skeleton A was found with the head in SSE. With it were a sword, two fibulas, three double buttons (one with an inlaid amber disc), a razor, a bronze knife, two bronze fish hooks, a tweezer, a small gold piece and a flint-strike-a-light associated [15]. Skeleton B was found with the head in NNV with a razor, an awl, a small piece of bronze, a flint-strike-a-light and a piece of pyrite associated with the deceased [15]. A previous aDNA analysis determined the skeleton (RISE 21) to be that of a male, with a: HG N1a1a1a2 [11].

In addition, there was a mandible (RISE 19) of a child in the grave. There is no information of where this was found. Therefore, it is difficult to say if this is part of the grave attire, like the remains of a cremated child in Egtved [16], or just part of the fill. A radiocarbon date demonstrates that the mandible from the child predates the two adult burials (1510-1420 cal BC). Skeleton A dates to 1510-1290 cal BC and skeleton B to 1430-1280 cal BC, indicating that the double burial probably occurred around between 1400 -1300 cal BC.

The strontium isotope values show that all three individuals suggest nonlocal origin, but they seem to come from different areas. The individual with the highest $^{87}Sr/^{86}Sr$ value, RISE 20, had an unusually low $\delta^{18}O$ value (-7.37 ‰) for the analysed material. The other two individuals had $\delta^{18}O$ values between -3 to -5 ‰ (carbonate values VPDB) which strengthens the hypothesis that they come from different areas.

The objects in the grave are all of Nordic style, possibly indicating that the deceased is from another area of the Nordic region. There is one similar bronze double button in Billegravsgård, Bornholm [17], which could indicate a connection to Bornholm, although

there are many other possibilities. If the mandible of the child was placed deliberately in the grave, it is possible that one of the two adults had brought the remains with them. It has been argued that in the case of the Egtved the discrepancy between the dendrochronological age of the coffin and the radiocarbon dates of the cremated bone relates to the old wood effect in cremations [18]. This might suggest the possibility that remains of a child could be a part of the living individual's life long after the child had died, as indicated by these two graves.

Kyndeløse (Møllehøj), Kirke Hylllinge parish Sb no 6, Volborg District, København County

Double passage grave

This grave was excavated in 1938 by Knud Thorvildsen. He only published a short account [19], but the site was extensively published by Ebbesen [20]. A mound, c. 18 m in diameter and 4 m high, contained two passage grave chambers, called the northern passage grave and the southern passage grave. Both tombs had clear stratigraphies with an upper and a lower layer, separated by layers of sand and crushed flint [20]. By their content of artefacts, the layers were suggested to be Late Neolithic and Middle Neolithic in date, respectively.

Large numbers of artefacts were found in both passage graves. The finds are described in detail in Ebbesen [20]. The upper layers had relatively few artefacts, among which flint daggers, fragments of pressure-flaked implements, and a Late Neolithic vessel may be noted. The flint daggers were eight in total and range typologically from Lomborg type I to type VI, covering the period from early Late Neolithic to the Early Bronze Age [20].

The lower layers were much more abundant in finds. Pottery from >75 vessels were found in the chambers and passages of both tombs, typologically ranging from Middle Neolithic A I to Middle Neolithic A V but with an emphasis on Middle Neolithic A IV pottery. In addition, large numbers of flint artefacts, bone and tooth pearls, and amber beads were found. Among

the flint artefacts were 14 thick-butted axes of which two hollow-edged, 8 thin-bladed thick-butted axes, 57 transverse arrowheads, 85 blades and 11 blade arrowheads of Pitted Ware type. Most of these artefacts can be dated to the Middle Neolithic A, but blade arrowheads and hollow-edged axes also indicate burials in the Middle Neolithic B period. The area in front of the passages had been disturbed, and almost no finds were made there [20].

A large amount of bone material was recovered at the excavation, but most of it was later discarded. Brøste et al. [10] estimated at least 73 individuals, of which 50 individuals were found in the lower layer and eight in the upper layer of the north chamber and passage. In the south chamber, they estimated there to have been at least 15 individuals in the upper layer, while no estimates were given for the lower layer, due to high fragmentation. Brøste et al. [10] 24 crania: PMD 13-29 (lower layer, all 17 from the north chamber) and PMD 89-95 (upper layer, 6 from the southern and 1 from the northern chamber).

The remaining bone material (mainly crania, mandibles and some long bones) was analysed by Pia Bennike [20]. She identified a minimum of 66 individuals in the remaining material, based on mandibles. She found remains of minimum 20 individuals in the north chamber lower layer, and 15 individuals in the upper layer. In the southern chamber, she identified at least 12 individuals in the lower layer and 14 individuals in the upper layer [20].

In the passage of the south chamber, one complete and one partially complete individual were found in the upper layer. Both were found in an extended position on their back. The complete individual (RISE 66, PMD 92), was interpreted as the last buried person. This is, however, contradicted by the dating to the Middle Neolithic B (Table 1 main text). Most other bones were commingled, and only a few partial articulations were noted.

Two crania showing signs of healed trauma (PMD 20 and 25) were dated by Fibiger et al. [21] to the early Middle Neolithic A. Both were found in the lower layer of the north chamber. Five individuals were sampled for aDNA by Melchior et al. [22].

Within the project, eight crania were sampled (RISE 60-66 and NEO867), representing different individuals. Four samples are from the lower layer, north chamber, two from the upper layer, south chamber and passage, one from the upper layer, north chamber, and one from unknown location. The radiocarbon dates show the sampled individuals from the lower layer to be from the Middle Neolithic B, i.e. from the Single grave/Pitted Ware phase (Table 1 main text). Only the two individuals dated by Fibiger et al. belong to the primary phase of the grave in Middle Neolithic A. These were not sampled by us, however. The three dates from the upper layer belong to the Middle Neolithic B, Late Neolithic and Late Neolithic/Early Bronze Age. The sealing of the lower layer thus seems to have occurred in the Middle Neolithic B rather than in the Middle Neolithic/Late Neolithic transition. Only one of the samples, RISE 61 (PMD17), had well-preserved aDNA, haplogroup J1c4 [11].

Langtved færgekro, Rye parish Sb no 112, Voldborg District, København County Gallery grave

The gallery grave was excavated in 1958, and was found to be built on an older kitchen midden (020611-112 [2]). The chamber was 2.75 m long and up to 0.75 m wide, and was covered by a partly destroyed mound. The human remains were disturbed and mostly disarticulated, as the western part of the cist had been disturbed [23]. Only one partly articulated individual was found in the cist (skeleton 1), of which only the lower part of the body remained. This was interpreted as the last individual buried. Human remains were also found outside the cist, from a probable clearance in prehistory [23]. In all, at least eight individuals had been buried in the cist. Of these, 4 individuals were sampled for this project

(RISE 14-17). The sampled individuals have been dated to the Late Neolithic II (Table 1 main text).

Trundholm mose, Højby parish sb no 586, Ods District, Holbæk County

Bog find

In 1940, two skeletons were found in a bog c. 3 km north of the Trundholm wagon (030405-586 [2]). They were found while ploughing. One of the skeletons, Trundholm mose II (RISE 276), was analysed for the project. The radiocarbon date indicated that the skeleton dates to the Late Bronze Age (800-540 cal BC). A previous aDNA analysis on RISE 276 showed that the deceased belonged to MtDNA haplogroup T2b and was male [11]. Other human remains have been found as close as c. 750 m from the Trundholm wagon (030405-331, 322 [2]).

Kolby Kås (Vormhøj), Kolby parish Sb no 42, Samsø District, Holbæk County

Barrow

A mound called Vormhøj in Kålby Kås was excavated by Christian Leif Vedbæk in 1960. Part of the mound had fallen into the sea. Remains of two inhumations and four cremations were found during the excavation [24, 25]. No artefacts were found in any of the graves, although a number of flint objects were found in the fill (Vedbæk 1963).

In the project the two inhumations were analysed. The central grave [24, 25] contained the remains of a child (RISE 18), and produced a radiocarbon date (1650-1510 cal BC) that places the burial in Period I. The second inhumation may have been an earlier flat grave[24, 25], and contained the remains of an adult (RISE 26). As expected, it is older than the central grave with its radiocarbon date of 1740-1560 cal BC.

Saltø Hovedgård, Karrebæk parish Sb no 22, Øster-Flakkebjerg District, Sorø County

Barrow

A mound containing five graves, including some scattered human remains, probably scattered due to animal activity, was excavated in 1998 [26].

The central grave (A38) held some animal bones and the remains of an adult individual (RISE 166) who measured 170 cm in the grave [26]. Grave A37 (RISE 168) was placed just above grave A38, and contained the remains of an adult who measured c. 170 cm in situ. It held a Bronze Age Period II tutulus and a bronze fragment [26]. Grave A35 (RISE 167) contained an adult [26]. Grave A34 contained a bronze double button dating to Bronze Age Period III, the handle of a flint dagger possibly of type VI, and previously, just above the grave, the tip of a bronze sword had been found. The skeletal remains of an adult were badly preserved [26]. The skeletal material in grave A32 was badly preserved but probably held the remains of an adult. Parts of two additional adults were found in the mound fill. In 1997 a further grave was found and dated to Bronze Age Period III [26].

In the report grave A38 was regarded as the oldest and grave A35 and A37 were seen as more or less contemporary [26]. The radiocarbon dates show that grave A38 (RISE 166) is probably the oldest, dating to Bronze Age Period I (1620-1500 cal BC). While A35 (RISE 167) and A37 (RISE 168) are more or less contemporary (1610-1420 cal BC and 1600-1410 cal BC) both belonging to Bronze Age Period I/II. Confirming the excavators' interpretation. A Bronze Age Period II date for A37 is more in with the style of the tutulus.

Hellested, Hellested parish Sb no 9, Stevns District, Præstø County

Flat graves

Four flat graves containing five individuals were found during the excavation of a shell midden in 1899-1900 by Hans Kjær [2, 10, 27, 28]. All five individuals were sampled.

Grave A (RISE 57, PMD 121/IV) was a flat grave below an almost oval stone pavement [9]. It contained the remains one adult individual, lying in hocker position. Under the left forearm

was a flint dagger type II A and by the left upper arm was a fragmented bone *Ringkopfnadel* (ring-headed pin; Ebbesen type 3; [9, 29].

Grave B (RISE 53) contained an adult individual lying extended in supine position, below a stone pavement. No artefacts were found [9].

Grave E was a flat grave below a low stone cairn. It contained two individuals, RISE 54 (PMD118/I) and RISE055 (PMD119/II), lying extended on their backs in opposite directions [27]. The grave contained two flint daggers of Lomborg type IB and one of type Ix [9, 27].

Grave F (RISE 56, PMD120/III) was a flat grave below an oval stone pavement [27]. It held an adult individual, lying in hocker position. The grave contained one fragmented bone pin [29].

All the graves date to the Late Neolithic I, with radiocarbon dates spanning from 2296 to 1985 cal BC. There are 96 BP years between the males in the double burial.

The ring-headed pin in grave A is of a common type for the Únětice culture [9, 29]. Lomborg [9] connects both grave A and E with the Únětice culture; in the case of grave E, this is due to the double burial in hocker position. This is not supported by Müllers account, however, as he describes them as lying in extended positions [27].

Strandfogedgård, Holtug parish Sb. No 29, Stevns District, Præstø County

Barrow

The mound was excavated by Henrik Thrane 1965-1966 [30]. The ploughed out mound contained an unusually large number of graves. The preservation of the bones was poor and not all of the graves contained human remains. The mound was probably built in two stages [30]. The RISE samples (RISE 13, 78 and 79) all came from the first mound stage.

Grave 15 [24] probably contained two individuals [30], one of which is likely to have been an adult based on the length of the skeleton drawn in the plan, and one child (RISE 13) [24]. Grave 15 is stratigraphically older than grave 13 (RISE 78), which is placed above grave 15 [30]. RISE 78 (grave 13, [24]) contained bronze fragments, a flint-strike-a-light and pyrite with organic remains around the finds, probably from a small bag placed to the west of the torso [24, 30]. The grave contained the remains of an adult. RISE 79 (grave 7, [24]) held no artefacts [24, 30], and the remains were of an adult.

The second mound phase contained three graves. Grave 1 (Ke 1357IA) contained artefacts belonging to Bronze Age Period II; a gold spiral, a bronze double button, an axe, a razor, a tweezer, an awl, a probable bronze belt hook, a fibula and flint strike-a-light and a flint arrowhead [24, 30].

None of the samples produced radiocarbon dates, but based on the artefacts in grave 1 and 5 the mound was built within a short time frame within Bronze Age Period II [30]. The flint strike-a-light has a probable Early Bronze Age date as most likely did all three analysed graves belong to the Early Bronze Age, Period I or II.

Ballehøj, Hasmark, Norup parish sb no 36, Lunde District, Odense County

Barrow

In 1949 Erling Albrectsen excavated sb no 36 and found a burial containing human remains, a dagger, two tutuli, a fibula, remains of a probable ring, a razor, a flint scraper, a fire strike-a-light, pyrite and remains of a ceramic vessel [17, 31]. The artefacts date the burial to MPII [17]. The analysis (RISE 170) showed a probable male aged 20-25 years.

Horne, Kimesbjerggårde, Horne lands parish Sb no 134, Salling District, Svendborg County

Barrow

Skeletal material, including teeth from a child, was found in a partly eroded mound (090412-134 [2, 32]). RISE 433 comprises human remains found in a grave when the mound was excavated. A flint dagger of Lomborg type VI was found with the remains (090412-134 [2, 32]), Mikkelsen (1989) writes that the flint dagger is Lomborg type III, but the later determination to Lomborg type VI which we find in Fund og Fortidsminder [2] is more accordance with the radiocarbon date. The grave contained an adult and a radiocarbon date places the deceased in Period I (1750-1560 cal BC).

Juelsbjerg, Nyborg parish, Vindinge District, Svendborg County

Gallery grave

P. Helweg-Mikkelsen excavated this gallery grave of Zealand type in a low, ploughed out barrow in 1939. The chamber contained at least 19 individuals [7]. Remains of at least five individuals were found in the cist, one of which was articulated and lay extended on its back [10, 29]. Outside the east end was a heap of bones from several individuals (~10)[10]. This heap was interpreted as resulting from a clearing of the chamber. Of the buried there were nine children and eight adults [7]. Seven crania were described by Brøste et al. [10], PMD 62-68. All these were determined as males.

The chamber contained a flint dagger type I [9], two flint arrow heads, an undecorated straight-walled beaker, a bone awl and two bone pins (one Ebbesen type 7 and one of indeterminable type) [7]. One of the arrowheads is a barbed and tanged arrowhead of west European Bell Beaker type [9, 33]. The flint dagger is of an early type [9], and the decorated pin seems to belong to the same phase. Bone pins of Ebbesen type 7 are mainly found in eastern Denmark, Scania and Västergötland, Sweden [9, 29].

Eight samples were taken from the grave (RISE 25, RISE 27 to RISE 33). Seven of the eight radiocarbon dates fall into the Late Neolithic I, and one in the Late Neolithic II (RISE 31).

The dates from the bone heap are slightly older than the datings from the chamber, supporting the idea of clearing.

We have no archaeological provenience of skeleton "V" (RISE 25) or skeleton VIII (RISE 28). Skeleton PMD68/VII (RISE 27), skeleton PMD66/V 2 (RISE 29) and skeleton PMD67/VI (RISE 33) were found in a bone heap, while Skeleton PMD62/I (RISE 30) Skeleton PMD63/II (RISE031) and skeleton PMD65/IV (RISE 32) were found in the chamber [10].

Sejerslev (Brunhøj), Sejerslev parish Sb no 47, Morsø Norre District, Thisted County Gallery grave

This gallery grave was excavated in 1925 by G. Hatt. The cist was 2.5 by 0.9 m large and dug into the ground. It contained some human remains as well as a flint dagger type Ix and a fragmentary bone pin [7].

Two adult individuals (RISE 67) and (RISE 68), were sampled. They are more or less contemporary and date to the Late Neolithic I: (RISE 67) 1950-1770 and (RISE 68) 2120-1890 cal BC.

Dommergården, Sælshyv', Vestervig parish sb. no 56, Refs District, Thisted County Barrow

This small ploughed out mound was excavated in 1974 by Jørgen Christoffersen. Three gallery graves (A-C) and remains of a possible fourth gallery grave (D) were found centrally below the mound. In the periphery of the mound, four Late Bronze Age urn graves were also found (E-I, [34]). Earlier, parts of a bronze sword had been found by the farmer.

RISE 105 is from grave A, a 1.7 by 0.4 m large gallery grave which lacked one cover stone and one side stone. The gallery grave contained remains of two adult individuals but no

artefacts [34]. In the bottom was a complete skeleton in extended position. The sampled individual lay on top of this in the western end of the chamber. It consisted of a cranium of an adult. In the same region were articulated upper and lower arm bones, possibly from the same individual. Dating failed on this sample, but a Late Neolithic date is likely.

Grave B, also a gallery grave, contained two broken flint daggers, a flint sickle, some flint splinters as well as some scattered and disarticulated human bones. Grave C, another gallery grave, contained a complete skeleton in extended position, measuring 165 cm in situ without the feet, disarticulated human bones, two parts of a flint dagger, some ceramic sherds and a bundle of six flint arrowheads [34].

Outside and to the west of grave B, some disarticulated skeletal remains from an adult were found. These were interpreted by the excavator as cleared out from grave B, which was supported by the find of the tip of a flint dagger (Lomborg type I) fitting with another fragment found in grave B [34]. He also suggested that the cranium and articulated arm found in grave A belonged to this individual. However, as the bones have not been studied in detail, this suggestion is not possible to substantiate.

Jestrup, Sønderhå parish Sb no 3, Hassing District, Thisted County

Barrow

The remains of an adult individual (RISE 104) were found in a stone cist in a mound. The grave contained a Rixheim type sword [35], a type that is mainly found in southwest Europe. A fibula and a bronze double button were also found in the burial [36, 37]. The sword is a non-local object, however the fibula and double button are Nordic types.

The radiocarbon analyses performed for the RISE project produced an earlier date than that indicated by the artefacts. The artefacts belong to Period III [36, 37], whereas the radiocarbon date indicated a Period I date for RISE 104, (OxA-28990, 3295 BP ±29 C13 -19.54; 1640-

1503 cal BC). A radiocarbon date on the femur, which was sampled for another project, had produced a different BP date (Lim-hb-143, UBA-31283 BP, 3114 ± 40, C13 -19.9, 1455-1269 cal BC); considering the marine intake 1430-1230 cal BC [38], this is more in line with the typological date.

It is suggested that this individual may have had the sword with him when he arrived in present-day Denmark, and acquired the fibula and the double button locally [36, 37].

Sønderhå, Sønderhå parish, Hassing district, Thisted Amt

stone cist/barrow

A small stone cist was found by a farmer. It contained a skeleton of an adult with the head to the west end and no objects. On the roof slab there were four urns with cremations. One of the urns contained some small bone objects [36]. The skeletal material from the cist was analysed for the project (RISE 12) and the radiocarbon date placed the find in the Early Iron Age (1-130 cal AD).

Sennels, Sennels parish Sb no 63, Hillerlev District, Thisted County

Barrow

This mound containing four graves was excavated in 1973 by Jørgen Christoffersen. Two graves contained no finds, grave 2 [36] contained a small ceramic cup and grave 4 [36] held a sword, bronze double button and a fibula, which dated the burial a date to the transition between Bronze Age Period III and Period IV [36].

The grave analysed within the Rise project is grave no 2 (in the AS registered as grave 1; RISE 107) and contains the remains of child and a small cup. The graves were renumbered after the remains were sent to the AS, therefore the numbers in the AS do not correlate to the number in the report. Grave number 3 (RISE 107) in AS is grave number 4 in the report and grave number 1 in AS is grave number 2 in the report [39]. The radiocarbon date failed, and

therefore we have no date for this grave, but based on the stratigraphy of the grave [36] the placement under grave D makes it likely to be a Bronze Age Period III grave, or at least belonging to the Early Bronze Age.

Nørhågård, Nørhå parish sb no 78, Hundborg District, Thisted County

Barrow

Mound sb no 78 was excavated in 1982 by Ann-Louise Haak. The barrow contained four graves. Grave N3 [36] contained the remains of an inhumation and a cremation. Grave N4 [36] and grave N5 [36] held burnt bones from cremations and the grave N9 [36] was an urn burial [18]. Within the project grave N3 (RISE 106) was analysed. The radiocarbon date on the inhumation dated the burial to 1260-1040 cal BC, late Bronze Age MPIII. The deceased was a probable male aged 25-35 years old.

Vorupørvej 16 (Høghs høj), Tilsted parish, Sb no 6, Hundborg District, Thisted County

Barrow

The ploughed out mound Høghs høj was partly excavated by Hans Andersen Kjær in 1912, and in 1978 completely excavated by Jens-Henrik Bech [36]. The mound contained five burials. Grave A (N2 in the report) contained a fibula, a bronze knife dating to Bronze Age Period III, and a flint scraper. Grave B (N3 in report) contained the cremated remains of an individual with a dagger and a pommel, a belt box, two arm-rings, a tutulus, a bracelet, four to five small bronze spirals, a pin, a double button, a sickle, a golden spiral-ring, 11 glass beads and part of a ceramic vessel. Grave C (N5) contained cremated bones and a bone pin, Grave D (N6) an inhumation dated to the Roman Iron Age and grave E (N26) a cremation dating to the pre-Roman Iron Age [36].

The teeth found in grave A (N2) have been analysed for the project (RISE 108). The teeth age the individual to be between 13 to 15 years old.

Tummelhøj, Rostrup parish Sb no 14, Hindsted District, Aalborg County

Gallery grave

A gallery grave placed in the centre of a partly ploughed out mound was excavated by G. Hatt in 1927 (120408-14 [2, 7]). The gallery grave contained disarticulated remains of at least five individuals and a flint dagger [7, 40]. The site is called Harehøjgårdhøj in Panums list.

Two individuals have been sampled for the project; RISE 58 an adult, dating to Late Neolithic II (1900-1740 cal BC); and another adult RISE 59 who date to Late Neolithic I (2120-1890 cal BC).

Sebber Skole, Sebber parish Sb no 12, Slet District, Aalborg County

Flat grave cemetery

Seven flat graves, containing at least eleven individuals were found during the excavation of a shell midden from the Ertebølle culture in 1908 by Rosenberg and Neergaard [28, 41, 42]. Ebbesen [41] argues that all the graves in the cemetery belong to Period I of the Bronze Age, based on the type VI flint dagger found with skeleton 10. It should be noted that no other graves contained artefacts, however. Six individuals were sampled for the project, RISE 47-52.

Grave 2 contained two individuals; skeletons No 2 and No 5. The grave contained no objects [41]. Skeleton No 2 (RISE 49) are an adult, lying in hocker position on his back [28, 41]. Below skeleton No 2 was skeleton No 5, another adult, lying in extended supine position [28, 41]. This individual was not sampled.

Grave 3 was covered by a stone packing, divided into two layers [41]. Between them was skeleton No 3, an adult individual. Under the lower layer were the remains from four individuals (skeletons Nos 6-8 and 10). Two of these were sampled (RISE047 and 048). Skeleton No 8 (RISE 47) an adult, lying in extended supine position [41]. Skeleton No 10

(RISE 48) was an adult, probably in a similar position although only the upper body remained [41]. Skeleton No 10 had been disturbed by the burial of skeleton No 8 which was thus stratigraphically younger [41]. A flint dagger of Lomborg type VI was found with the former skeleton [41, 42].

Grave 4 contained a skeleton an adult in hocker position on its right side (skeleton No 4; [41]). There may also have been other individuals in the grave, as there are skeletal fragments from several individuals of different age groups stored with grave 4 at the AS. This despite Ebbesen [41] only mentions skeleton No 4. Rosenberg [42] mentioned skeleton No 4 as well as remains from other individuals. From this grave we have two samples: RISE 52, skeleton No 4, and RISE 51.

The last skeleton that was sampled in the project is from grave 6 (RISE 50). This grave contains the remains of a two individuals an adult and a child. The sampled individual was the adult.

Only RISE 47 yielded aDNA information and the genetic information determines the sex to male and the HG is I [11].

The radiocarbon dates disprove Ebbesen's [41] hypothesis that all the deceased here belonged to Bronze Age Period I. The radiocarbon dates range from Late Neolithic II (RISE 49) to Bronze Age Period II (RISE 47). The two radiocarbon dated skeletons in grave 4 have virtually identical dates, with only 18 BP years difference. Skeleton No 8 (RISE 47), which was stratigraphically younger than skeleton No 10 (RISE 48), though that cannot be seen in the calibrated dates only in the BP date. RISE 48 and 47 dates to the first half of Period II (1500-1320 BC).

Debel, Fur parish Sb no 12, Harre District, Viborg County

Barrow

Only one grave is known from the mound, placed centrally in the barrow. The grave contained the remains of an individual that in the grave measured 171 cm. On the left shoulder were 47 amber pieces and by the right hip a belt hook was placed. In the foot end of the grave were burnt bones which probably had been wrapped in textiles as some textile fragments were found with them [43]. The majority of the bones were removed from their box in Panum and could therefore not be analysed within the project. The radiocarbon date 1530-1420 cal BC (RISE 76) places the burial in Period PII.

Debel, Fur parish Sb no 24, Harre District, Viborg County

Barrow

Five burials were excavated in 1931 by Johannes Brøndsted. Grave A was a disturbed stone cist that held a stone with rock-art, a number of cup-marks, a cross-shaped form and some other shapes, and skeletal remains of an adult in extended position on his back. No artefacts were found [37, 43-45]. Grave B, a small stone cist, contained a few bones of a child, without grave goods [43]. Grave C was also a small stone cist, which held the remains of a child and an amber bead [43]. Grave D and E were in the form of small circular stone settings that contained no objects [43]. Grave F held the remains of an inhumation and a double button that is now lost; based on a drawing it probably belonged to Period III [43].

This is called Sb no 25 in Aner et al. [43], but in Fund og Fortidsminder is Sb. 24. In AS's archive the site is called Bakkehøiene, The grave numbers in AS and Aner et al. are also different, i.e. what is numbered in the AS's list as grave C is grave A in Aner et al. [43].

Only grave A was sampled (RISE 23). The skeleton is of a male aged 20 to 25 years, who was a minimum of 176 cm tall. The radiocarbon date is 1450-1290 cal BC, placing the burial in late Bronze Age Period II.

Hverrehus, St Torup, Ulbjerg parish sb no 163, Rinds District, Viborg County

Flat graves

While digging on a stretch of beach in a heap of stones among seashells south of Hverrehus a worker unearthed a large number of bronzes [2, 46]. The site was later excavated and contained five flat graves [43].

Grave A was a rich adult female burial, who had worn a corded skirt ([37, 43, 46-48]. Grave B, another likely female burial, contained a neck collar and small ceramic beaker ([37, 43, 46]. Grave C was discovered in 1919 and there is no detailed information about the find other than that it was found in the same shell bank as grave A [46], and that it contained a small belt-plate. Grave D contained the remains of a sword, a fibula and a bracelet [43]. Broholm [46] argued that Grave C and D are just one grave, containing the remains of another female burial. Grave E (RISE 282) was found 10 m south of grave C and contained the remains of a child and a tutulus [43].

The graves can be typologically dated to MPII [43]. This date is confirmed by the radiocarbon date conducted for the project on the remains from grave E (RISE 282), which calibrated with 2 sigma produced the year 1500-1380. The individual buried in grave E is a child. The tutulus is of the same type that was found in the rich female grave A.

Øster Herup, Lihme parish Sb no 124, Rødding District, Viborg County

Barrow

This small mound with a small stone cist was excavated by Mette Iversen in 1974. The cist contained a partly preserved skeleton, which, based on the coffin, was dated to the Early Bronze Age [49]. In Fund og Fortidsminder 131005 Sb no 124 no information is provided about this excavation location, but based on the report and the map in the report it must be the same mound, as part of the information is correct.

The skeleton (RISE 460) is radiocarbon dated to 1610-1420 cal BC, i.e. most likely Bronze Age MPII. The remains are from an undetermined adult aged 30 to 35 years' old**.**

Bustrup, Ramsing parish Sb no 22, Rødding District, Viborg County

Barrow

Hans Kjær examined the mound in 1916 and 1917 after a farmer encountered the central grave of the barrow. Two burials were found during the investigation [43, 50]. The central grave (grave A; RISE 281) was rich, containing a bronze neck ring, belt-plate, metal-hilted dagger and chase, numerous bronze tubes, four beads made of animal teeth (one wild boar and three dog), two ceramic beakers and some textile remains [37, 43, 51]. The textile remains are from a blouse or a blanket and a corded skirt [51]. The technique used in the starting border of the corded skirt is similar to the found in Ølby, Zealand [52]. The second grave in the mound (grave B) contained a sword blade and a pommel, a dagger, a flanged axe, a fibula, a tweezer and a flint strike-a-light [37, 43]. Both graves belong to Period II [37, 43]. Grave A (RISE 281) is dated to 1440-1280 cal BC.

Gjessinggård, Tvede parish Sb no 37, Nørhald District, Randers County

Flat grave

This grave containing human remains of an adult and a bronze fibula was excavated by Neergaard in a shell midden in 1917 [37]. The grave has been analysed within the project (RISE 22). The fibula is a cruciform fibula [37], which dates the grave to MPIII, and is consistent with the radiocarbon date (1410-1230 cal BC), which places the burial in the late Bronze Age MPII and early MPIII.

Falshøj, Mariager parish Sb no 172, Omsil District, Randers County

Dolmen/gallery grave

Chance find reported by the farmacist Mikkelsen in 1880 [10, 53]. A chamber of uncertain form was discovered, c. 2.35 m long and 1.55 m wide (E-W), surrounded by a mound. The chamber had 6 uprights and a single large capstone. A kind of entrance was noted in the eastern end. The description of the chamber is vague and could fit a dolmen or a gallery grave. The remains of at least 11 people were noted in the chamber [10]. Few artefacts were recovered; a flint dagger of "late type" and an undecorated clay cup.

Three adult individuals were sampled from this grave: RISE 69 (PMD59), RISE 70 (PMD60), RISE 71 (PMD57), All three individuals were dated to the Late Neolithic (Table 1 main text). A further individual, PMD58, was dated by Fibiger et al. [21], also to the Late Neolithic. This individual showed cranial trauma in the form of ante-mortem fracture to the zygomatic (cheek bone) [21]. RISE 71 has previously [11] been analysed for aDNA, the result showed a female with haplogroup H3b.

Gjerrild, Gjerrild parish Sb no 9, Randers Nørre District, Randers County

Gallery grave of Bøstrup type

The grave was excavated in 1956 by C. L. Vedbaek, and is still the grave with the most substantial human bones dating to the Danish Single Grave Culture [54-56]. The grave chamber had been disturbed but the outline could be reconstructed. It was 2.8-3 m long and 1.6-1.7 m wide in the broader northern end, while it was only ca 1 m wide in the southern gable, where the entrance was found.

The artefacts in the chamber were of SGC types and included two SGC vessels, three blade arrowheads of D-type, a thin-bladed flint axe and two amber beads. Some artefacts could be associated with specific individuals, see below. In spite of partial destruction of the chamber, burials and artefacts on the chamber floor were largely undisturbed.

Osteological analyses were carried out by Balslev Jørgensen and complemented by Bennike [7, 55], and teeth have been analysed by Alexandersen [57]. The grave contained at least ten individuals: six adults, one juvenile and three children. Several of the skeletons were articulated, but some were also more or less disturbed and disarticulated. Two skeletons were in extended supine positions, while two were in hocker positions. According to Ebbesen [55] Balslev Jørgensen has identified several individuals, numbered 1-9:

Individual 1 (RISE 1283) an adult placed in hocker position, dated to the MN B. Individuals 2-4: scattered fragments of 3-4 individuals. Not sampled. Individual 5 (RISE 1281), a child, dated to the Late Neolithic. Individual 6 (RISE 73a, 1282) an adult, with type D arrowhead in chest bone, dated to the MN B. Individual 7 (RISE432, 1415) an adult, D-type blade arrowhead and amber bead by the right hip. It was suggested by Vedbaek [54] to be the last buried person. However, this is contradicted by the dating to the MN B. Individual 8 (RISE 1280) a child, dated to the MN B. A disarticulated mandible was also sampled, RISE 72. This was dated to the Early Bronze Age.

Nybøl, Hjordkjær parish sb no 64, Rise District, Åbenrå County

Barrow

A farmer at Nybøl, in South Jutland, uncovered an oak-log coffin in the summer of 1888 while demolishing a large mound situated on his land [58]. A substantial number of textile remains were found in the oak-log coffin. Two non-textile artefacts were also discovered: a horn comb and a razor, placed at the right hand [58, 59]. The skeleton (RISE 326) has previously been claimed to be that of a male, c. 175 cm tall [10]. The deceased has also been claimed to be 30 years old and 170 cm based on information published straight after the oak-log coffin was found [60]. Both the razor and the horn comb seem to have been heavily used [61]. The razor with a horse head is a common type of razor in Scandinavia [62]. Only one similar comb is known to the authors in the Scandinavian material, a horn comb found in

Stora Köpinge, Scania Sweden. The grave contained a sword and some textile remains as well as the comb [63]. The material in the Stora Köpinge burial is similar, but not identical, and it is earlier than the Nybøl burial since it dates to Bronze Age PII [64].

The oak coffin has been dated by dendrochronology to 1266 cal BC -11/+20 years, giving the time span 1277-1246 cal BC (Christensen 2006, 212). The artefacts date to Bronze Age Period III, which is in accordance with the dendrochronological date. The sample RISE 326 was radiocarbon dated for the project (1300-1120 cal BC), which agrees with the dendrochronology date.

References:

1. Drews F. Ballerosen, Dråby s., Horns hrd., Fred. Amt, mb 2926, sb. 320. Archive National Museum Copenhagen: 1958.
2. Stewart BW, Capo RC, Chadwick OA. Effects of rainfall on weathering rate, base cation provenance, and Sr isotope composition of Hawaiian soils. Geochimica Et Cosmochimica Acta. 2001;65(7):1087-99. PubMed PMID: ISI:000167957500007.
3. Baudou E. Die regionale und chronologische Einteilung der jüngeren Bronzezeit im Nordischen Kreis. Stockholm: Almgren & Wiksell; 1960.
4. Stumann Hansen S, Larsen A-C. Store Havelse Strand. Beretning for amtsarkæologisk kontor ved Gilleleje musum arkeæologiske udgravning på lokaliteten Store Havelse Strand, Ølsted sogn, Strø herred, Fredriksborg Amt i 1986, GIM 3161. Archive National Museum Copenhagen: 1992.
5. Kastholm OT. Bronzealderbadene fra Varpelev og Vesterso i et europaisk perspektiv. Aarbøger for nordisk Oldkyndighed og Historie. 2016;2015:59-115.
6. Koch E, Bennike P. Hove å, Københamn Amt. Arkæologiske udgravninger i Danmark. 1995;1995:282.
7. Ebbesen K. Danske hellekister fra stenalderen. Aarbøger for Nordisk Oldkyndighed og Historie. 2007;2004:7-62.
8. Nielsen HA. Fortsatte Bidrag til vort Oldtidsfolks Anthropologi. De seneste 5-6 års skeletfund fra stenaldergrave og særligt de sidste 10-12 års skeletfund fra Jernaldergrave. Aarbøger for Nordisk Oldkyndighed of Historie 1915;1915:275-370.
9. Lomborg E. Die Flintdolche Dänemarks. Studien über Chronologie und Kulturbeziehungen des südskandinavischen Spätneolithikums. Copenhagen: Det Kgl. Nordikse Oldskriftselskab; 1973.
10. Brøste K, Balslev Jørgensen J, Becker CJ, Brøndsted J. Prehistoric Man in Denmark. A Study in Physical Anthropology. Copenhagen: Einar Munksgaard Publishers; 1956.
11. Allentoft ME, Sikora M, Sjögren K-G, Rasmussen S, Rasmussen M, Stenderup J, et al. Population genomics of Bronze Age Eurasia. Nature. 2015;522:167. doi: 10.1038/nature14507.
12. Liversage D. En hellekiste ved Gerdrup, Københavns Amt. Aarbøger for nordisk Oldkyndighed og Historie. 1964;1964:32-62.
13. Nielsen OV, Alexandersen V. Antropologisk og odontologisk undersøgelse af de sen-neolitiske skeletrester fra Gerdrup. Aarbøger for Nordisk Oldkyndighed of Historie 1965;1964:63-71.

14. Aner E, Kersten K. Die Funde der älteren Bronzeziet des nordischen kreises in Dänemark, Schleswig-Holstein und Niedersachsen: Fredriksborg und Københavns Amt Neumünster: Karl Wachholz Verlag; 1973.
15. Vedbæk CL. Fundberetning 4319, Karlstrup. Archive National Museum Copenhagen: 1987.
16. Thomsen T. Egekistefundet fra Egtved, fra den ældre Bronzealder. Nordiske Fortidsminder 1929;II(4):165-214.
17. Aner E, Kersten K. Die Funde der älteren Bronzeziet des nordischen kreises in Dänemark, Schleswig-Holstein und Niedersachsen: Bornholms, Maribo, Odense und Svendborg Amter Neumünster: Karl Wachholz Verlag; 1977.
18. Hornstrup KM, Olsen J, Heinemeier J, Thrane H, Bennike P. A New Absolute Danish Bronze Age Chronology as based on Radiocarbon Dating of cremated Bone Samples from Burials. Acta Archaeologica. 2012;83(1):9-53. doi: doi:10.1111/j.1600-0390.2012.00513.x.
19. Thorvildsen K. Dobbeltjættestuen på Kyndeløse Mark. Fra Nationalmuseets Arbejdsmark 1939;1939:19-28.
20. Ebbesen K. Danske Jættestuer. Copenhagen: Attika; 2009.
21. Fibiger L, Ahlström T, Bennike P, Schulting RJ. Patterns of Violence-Related Skull Trauma in Neolithic Southern Scandinavia. American Journal of Physical Anthropology. 2013;150:190–202.
22. Melchior L, Lynnerup N, Siegismund HR, Kivisild T, Dissing J. Genetic Diversity among Ancient Nordic Populations. PLOS ONE. 2010;5(7):e11898. doi: 10.1371/journal.pone.0011898.
23. Voss O. Langtved Fargekro, Rye sogn, Voldborg, Herred, Kbenhavn Amt journal nummer 848/58. Panum Archive, Copenhagen: 1958.
24. Aner E, Kersten K. Die Funde der älteren Bronzeziet des nordischen kreises in Dänemark, Schleswig-Holstein und Niedersachsen: Holbæk, Sorø und Præstø Amter. Neumünster: Karl Wachholz Verlag; 1976.
25. Vedbæk CL. Sagens Papir lagt til Beretningen om Undersøgelsen af Bronzealdershøjen Kolby Sb. No 42. Archive National Museum Copenhagen: 1963.
26. Høst Madsen L, Roland T. Saltø Hovedgård, Etapp II – 1998 NÆM 1997:103, Næstved Museum 2000. Archive National Museum Copenhagen: 2002.
27. Müller S. Flintdolkene i den nordiske stenalder. Nordiske Fortidsminder. 1902;1:125-80.
28. Nielsen HA. Yderligere Bidrag til Danmarks Stenalderfolks Anthropologi. Aarbøger for Nordisk Oldkyndighed of Historie. 1911;1911:81-105.
29. Ebbesen K. Spätneolithische Schmuckmode. Acta Archaeologica 1995;55:201-79.
30. Thrane H. Overpløjet høj Strandfogdegården Sb. 29, Holtug s., Stevns h., Præstø a. Undersøgt 1965-1966. 504/66 Archive National Museum Copenhagen: 1966.
31. Albrectsen E. Egekistegrav fra den ældre bronzealder: udgravet i Ballehøj under matr. No. 26g af Hasmark by, Norup sogn, Lund herred, Odense amt. 1949.
32. Mikkelsen H. 152. Kimesbjerggård, 09.04.12 Horne. Arkæologiske udgravninger i Danmark 1989;1988:127.
33. Nicolas C. Flèches de pouvoir à l'aube de la métallurgie de la Bretagne au Danemark (2500-1700 av. n. è.). Leiden: Sidestone Press; 2019.
34. Christoffersen J. Rapport, Vestervig s., Sb.no 56. St.. no. 11.06.12. Journ. No. 1165. Museet for Thy of Vester Hanherred Archive: 1974.
35. Schauer P. Die Schwerter in Süddeutschland, Österreich und der Schweiz. 1, Griffplatten-, Griffangel- und Griffzungenschweter. München: C.H. Beck; 1971.
36. Aner E, Kersten K, Willroth K-H. Die Funde der älteren Bronzeziet des nordischen kreises in Dänemark, Schleswig-Holstein und Niedersachsen: Thisted Amt Neumünster: Karl Wachholz Verlag; 2001.
37. Broholm H-C. Danmarks bronzealder: Kultur og folk i den äldre bronzealder. Copenhagen: Arnold Busck; 1943.
38. van der Sluis LG. Investigating palaeodietary changes from the Mesolithic to the Viking Age in the Limfjord area in northern Denmark: Queen's University Belfast; 2017.

39. Bech J-H. Vdr. analyse af tandmateriale fra æ. bronzealdergrav i Sennels sogn AS 7/72. Panum Archive, Copenhagen: 1987.
40. Hatt G. Ardm, 4 Okt. 1928 diar no 460/27. Archive National Museum Copenhagen: 1927.
41. Ebbesen K. En gravplads fra ældre bronzealder ved Sebber Skole. Gravfund med flintdolke af type VI. Aarbøger for Nordisk Oldkyndighed og Historie. 2005;2002:7-37.
42. Rosenberg G. Skaldynge med Grave paa Sebber Skoles Legeplads, Sebber Sogn, Slet Herred, Alborg Amt. Archive National Museum Copenhagen: 1908.
43. Aner E, Kersten K, Willroth K-H. Die Funde der älteren Bronzeziet des nordischen kreises in Dänemark, Schleswig-Holstein und Niedersachsen: Viborg Amt Neumünster: Karl Wachholz Verlag; 2008.
44. Brøndsted J. Ein neugefundener Schalenstein aus Fur, Jylland. Acta Archeologica. 1931;1931(2):203-6.
45. Glob PV. Helleristninger i Danmark: Rock carvings in Denmark. Copenhagen: Jysk Arkæologisk Selskab; 1969.
46. Broholm H-C. Tre kvindegrave fra den ældre bronzealder fra Gjedsted sogn. Aarbøger for Nordisk Oldkyndighed of Historie 1940;1940:117-40.
47. Bergerbrant S. Ordinary or extraordinary? Redressing the problem of the Bronze Age corded skirt. Current Swedish Archaeology 2014;22:73-96.
48. Randsborg K. Bronze Age Textiles: Men, Women and Wealth. London: Bristol Classical Press; 2011.
49. Iversen M. Fire slags grave. Museerne i Viborg. 1975;1975:62-3.
50. Kjær H. I høj, Bustrup, Ramsinge S., Rødding H., Viborg A. 1916. Archive National Museum Copenhagen: 1917.
51. Bender Jørgensen L. Forhistoriske textile i Skandinavien. Prehistoric Scandinavian Textiles. Copenhagen: Det Kgl. Nordiske Oldskriftselskab; 1986.
52. Fossøy SH, Bergerbrant S. Creativity and Corded Skirts from Bronze Age Scandinavia. The Journal of Cloth and Culture. 2013;11(1):20-37. doi: 10.2752/175183513X13588738654819.
53. Nielsen HA. Bidrag til Danmarks Forhistoriske Befolknings (særligt Stenalderfolkets) Anthropologi. Aarbøger for Nordisk Oldkyndighed of Historie 1906;1906:237-318.
54. Vedbæk CL. Et usedvanligt stenalders gravfund paa Djursland. Fra Nationalmuseets Arbejsmark. 1957;1957:75-82.
55. Ebbesen K. Nordjyske gravkister med indgang. Bøstrup-kisterne. Aarbøger for Nordisk Oldkyndighed og Historie. 1985;1983:5-65.
56. Hübner E. Jungneolitische Gräber auf der Jütischen Halbinsel. Typologische und chronologische Studien zur Einzelgrabkultur I-II. Copenhagen: Det kongelige nordiske oldskriftselskab; 2005.
57. Alexandersen V. Tandforholdene i Enkeltgravstid/stridsøksetid. In: Larsson L, editor. Stridsyxekultur i Sydskandinavien: rapport från det andra Nordiska symposiet om stridsyxetid i Sydskandinavien, 31 X-2 XI 1988. Institute of Archaeology Report Series Lund: Lund University; 1989. p. 169 - 80.
58. Boye V. Fund af egekister fra bronzealderen i Danmark. Aarhus: Wormianum; 1896[1986].
59. Aner E, Kersten K. Die Funde der älteren Bronzeziet des nordischen kreises in Dänemark, Schleswig-Holstein und Niedersachsen: Nordslesvig - Syd Neumünster: Karl Wachholz Verlag; 1981.
60. Jensen J. Manden i kisten hvad bronzealderns gravhøje gemte. Copenhagen: Gyldendal; 1998.
61. Bergerbrant S, Jørgensen LB, Fossøy SH. Appearance in Bronze Age Scandinavia as Seen from the Nybøl burial. European Journal of Archaeology. 2013;16(2):247-67. doi: 10.1179/1461957112Y.0000000026.
62. Kaul F. The Nordic razor and the Mycenaean lifestyle. Antiquity. 2013;87(336):461-72. Epub 2015/01/02. doi: 10.1017/S0003598X00049061.
63. Oldeberg A. Die ältere Metallzeit in Schweden. Stockholm: Kungl. Vitterhets historie och antikvitetsakademien; 1974.

64. Håkansson I. Skånes gravfynd från äldre bronsålder som källa till studiet av social struktur. Lund: Lund University; 1985.
