# Supplementary material for: Mapping human mobility during the third and second millennia BC in present-day Denmark
Source: PLoS One. 2019 Aug 21;14(8):e0219850. doi: 10.1371/journal.pone.0219850 (PMC6703675; doi:10.1371/journal.pone.0219850)
Supplement: S1 Table — (PDF) [file pone.0219850.s002.pdf]

S2 Table. Overview of the anthropological data.

Rise-no.	Skeleton-no.	Museum-no.	Sample	Sex	Age (in years)	Femur length (cm)	Stature measured in grave or previously calculated (Trotter and Gleser 1958)	Skeletal pathology and traits	Dental pathology and traits
RISE 12	skeleton	NM 418/31, NM B 12304	LRP2	female	18-20			Inflammatory changes on skull: porotic hyperostosis interna on left parietal (medial). Long and slender long bones.	
RISE 13	grave 15 e	NM 504/66	LLM1	nd	*c.* 8 years				Shoveling of almost all incisors, LLP1 has three roots.
RISE 14	child #2	NM 848/58	URm2	nd	*c.* 6			Signs of physiological stress/vitamin deficiency: cribra orbitalia in left orbit.	
RISE 15	#5	NM 848/58	LRM2	male	20-30				
RISE 16	#4 (+ #7)	NM 848/58	LRP2	male	25-40	49.5	(179 cm)	"Riding facets" on left caput femoris. Porosity in palate.	
RISE 17	child #3	NM 848/58	ULm1, URm2	nd	*c.* 4			Signs of physiological stress/vitamin deficiency: cribra orbitalia, tall for the age.	
RISE 18	"Central grave"	NM 405/60	LLM1	nd	*c* . 8-9				
RISE 19	Mound 4, northern skeleton # 2	NM 1 469/65	LRm2	nd	*c.* 7-8				
RISE 20	Mound 4, southern skeleton	NM 1 469/65	LLM3	male	25-40	50.5	(181.5 cm)	Slight osteophytes on thoracic vertebrae. Previous analysis from 1965 by Balslev Jørgensen mentions osteoarthritis in right shoulder and elbow joint but could not be confirmed due to poor preservation. Long, slim long bones. Marked muscle attachments on femurs.	Tooth pick furrows.

RISE 21	Mound 4, northern skeleton #1	NM 1 469/65	LRM2	male	20-25	(177 cm)	Slight degenerative change in dens axis of C2.	Possible tooth pick furrow on premolars (URP1 and URP2). Assymetric dental wear. Mostly on right side. Genetic variations of molar roots (radix entomolaris).
RISE 22	Skaldynge 2	NM B 10603	premolar	female	30-40			AM tooth loss of seven teeth in mandible. LEH (three bands on mid crown) on ULP1. Slight calculus.
RISE 23	grave C	NM B 12535	LRP2	male	20-25		Long slim skeleton.	
RISE 24	Ballermosen	NM 959/58	ULI1	male	50+		Very robust skeleton.	
RISE 25	V	NMA38669-75	LLP2	nd	25-35			Caries lesions in three teeth. Calculus.
RISE 26	"vormhøj"	NM 405/60	Unid. tooth	male	35+		Osteoarthrisis in cervical and lumbar vertebrae.	
RISE 27	PMD 68 (VII)	NMA38669-75	LRP1	male?	35-45			AM tooth loss and inflammation in several alveoli in maxilla. Crowding of canines.
RISE 28	VIII	NMA38669-75	LRP2	nd	*c* . 14-15			Severe calculus deposits on left premolars and molars in mandible and maxilla. Possibly the prefered side when chewing.
RISE 29	PMD 66 (V)	NMA38669-75	ULI2	male	35-50			LEH.
RISE 30	PMD 62 (I)	NMA38669-75	URP2	male	40+			Slight LEH. Heavy tooth wear in maxilla, less in mandible (malocclusion?). Slight calculus.
RISE 31	PMD 63 (II)	NMA38669-75	ULP2	female	20-30			LEH on several teeth (incisors, canines and premolars (2-3 lines).
RISE 32	PMD 65 (IV)	NMA38669-75	LRP2	female	25-35			Several caries lesions. Calculus.
RISE 33	PMD 67 (VI)	NMA38669-75	LLP2	male?	20-30			Some calculus. Slight LEH on incisors and canines.
RISE 34	II, PMD 97	NMA 27037-46	LRP2	female	25-40			
RISE 35	XI, PMD 105	NMA 27037-46	LRM3	male?	20-25			
RISE 36	PMD 96-155	NMA 27037-46	LRP1	male?	20-25			Slight calculus. Slight periodontitis.
RISE 37	VII, PMD 101	NMA 27037-46	URP1	female	20-30+			Slight calculus, slight LEH on canines.
RISE 38	I, PMD 96	NMA 27037-46	LLP2	male	35-45		Largening of condylar foramen canal (skull base), possibly a normal variation.	Moderate calculus.
RISE 39	IV, PMD 99	NMA 27037-46	LRC	female?	35-45		Possible healed blunt force trauma with no penetration into diploe.	
RISE 40	X, PMD 104	NMA 27037-46	URC	male	30-40		Evidence of physiological stress/vitamin deficiency: healed cribra orbitalia and porosity in palate.	Slight LEH on incisors and canines. Calculus. Slight periodontitis.
RISE 41	V, PMD 100	NMA 27037-46	LLP2	female?	30-40			Abscess. Periodontitis. Calculus. LEH on incisors and canines.

RISE 42	III, PMD 98	NMA 27037-46	URC	male	40+		LEH on canines. Calculus. Abscess on buccal side of LLP2. Heavy dental wear. Crown-shape of molars appear flattened.
RISE 43	VIII, PMD 102	NMA 27037-46	LRP2	male	40+		
RISE 44	IX, PMD 103	NMA 27037-46	URC	male	45+		Inflammation at aveolar margins. Significant ante mortem tooth loss. Heavy dental wear
RISE 45	bag 14-22-24	NM 1 603/63	LLM1	nd	*c* . 12-16		Shovel-shaped insicors.
RISE 46	bag 11-12-26	NM 1 603/63	LLP1	nd	adult		
RISE 47	grave 3, skeleton 8, N358,	NM A 24697	LRP2	male	25-35		Premolars in mandibula with three roots.
RISE 48	"3, skeleton 10" N360 "box 137"	NM A 24697	ULP1	male?	40-50		Severe caries.
RISE 49	"grave 2, skeleton 2" N348	NM A 24697	LLP1	male	25-35		LEH (3-4 lines) on incisors, canines and first premolars in mandible and maxilla. Slight calculus. Agenesis or AM loss of LRP1 or LRP2.
RISE 50	grave 6, N318	NM A 24697	LLP1		adult		
RISE 51	"Gr. 4"	NM A 24697	premolar	nd	18-25		
RISE 52	grave 4, skeleton 4 " .85" (N375)	NM A 24697	premolar	male	20-30		Caries in LRM1 and abscess in lower right mandible at LRM2. LEH on LRC.
RISE 53	grave B	NM A 18183-84	URP2	female?	40-45		Periodontitis, AM tooth loss of LRP2 and LLP2 (could also be agenesis). Caries in lower molars.
RISE 54	PMD 118 "I", grave E	NM A 18185	URP1	male?	21-23	Ossicles on the parietal bones -a nonmetric trait.	Slightly prognatic, crowding of teeth.
RISE 55	PMD 119 "II", grave E	NM A 18185	LLC	male	20-25		Slightly prognatic. Periodontitis. Molars in maxilla have rhombic shape.
RISE 56	PMD 120 "III", grave F	NM A 18188	LLP2	male	*c* . 20	Very dense skull. Healed cribra orbitalia, porosity in the palata. Signs of porotic hyperostosis (skull). Porotic hyperostosis may be related to density of skull as often seen in stone age skulls.	
RISE 57	PMD 121 "IV", grave A	NM A 18183-84	LLP1	male	*c* . 18	Healed cribra orbitalia, spina bifida (congenital defect).	LEH on incisors. Slight shoveling of incisors.
RISE 58	box 31	NM A 33629	ULP1	nd	20-30		LEH on incisors and canines (several lines).
RISE 59	nummer 2 (box 45)	NM A 33629	ULP2	nd	35-45		AM tooth loss of ULM1 with partical healing of aveolar.
RISE 60	XVIII, PMD 90, southern chamber, cranium g, field 1, øvre lag (SN)	NM A 38067, NM 205/38	ULP2	female	30-40	Occipital bossing /low calvarium -could be taphonomic.	Some calculus. Agenesis of URM3 and ULM3. Small teeth.

RISE 61	V, PMD 17, northern chamber, cranium o, field 6, lowest layer (MN)	NM A 38067, NM 205/38	URP2	male	20-(25)	Healed cribra orbitalia.	URM3 is not in occlusion. Small teeth.
RISE 62	IV, PMD 16, northern chamber, cranium y, field 2, lowest layer (MN)	NM A 38067, NM 205/38	LLP2	female	25-35		Slight shoveling of mandibula canines. Small teeth.
RISE 63	XXIV, PMD 93, northern chamber, cranium C, field 6, top layer (SN)	NM A 38067, NM 205/38	URP1	female	20-25	Healed cribra orbitalia.	Calculus. Small teeth.
RISE 64	III, PMD 15, northern chamber, cranium A(?), field 8, lowest layer (MN)	NM A 38067, NM 205/38	ULP1	nd	25-35		Agenesis of URM3 and ULM3. Small teeth
RISE 65	X, PMD 21, northern chamber, cranium g, field 2, lowest layer (MN)	NM A 38067, NM 205/38	ULP1	female	20-30		Small teeth.
RISE 66	XXI, PMD 92, southern chamber, cranium a, field x, top layer (SN)	NM A 38067, NM 205/38	ULP2	male?	30-40		LEH on LRC. Agenesis of URM3.
RISE 67	small box no ID	NM A 32919-20?	LRP2	nd	20-35		
RISE 68	"brunshøj" big box, kranium 21	NM A 32919-20	ULP1	nd	18-25		Possible agenesis of URM3.
RISE 69	III, PMD 59	NM A 4193	URP2	female	30-35	Unilateral concha bullosa.	
RISE 70	IV, PMD 60	NM A 4193	M2	male	25-35	Unilateral concha bullosa.	URM3 has 6 cusps and has a rhombic shape.
RISE 71	I, PMD 57	NM A 4193	URC	female	25-35	Less pronounced but still unilateral concha bullosa (normal nasal variation). Ossicles at bregma.	Moderate calculus.
RISE 72	disarticuated mandible found w. skeleton nr. 6	NM 704/56	LRP1	nd	adult		
RISE 73 a (1282)	Skeleton nr. 6	NM704/56	M1	male	25-35	Arrowhead in sternum	Slightly shovel shaped incisors.
RISE 1280		NM704/56	Unid. tooth	nd	subadult		
RISE 1281		NM704/56	Unid. tooth	nd	subadult		
RISE 1283	skeleton nr. 1	NM 704/56	LLC	female	21-30		Slightly shovel shaped incisors.

RISE 432	skeleton nr. 7	NM 704/56 no. NM A 44578)	LLM1	male	35-50			Trepanation on left parietal. Periostitis on medial and lateral side of lower legs, in particular left fibula. Large and robust skeletonon with marked muscle attachments.	
RISE 76	Fur Museum 12 x217	Jnr. 496/62	premolar	male?	adult				
RISE 78	grave 13	NM 504/66	LLP2	nd	adult				
RISE 79	grave 7	NM 504/66	M1	nd	25-35				Calculus.
RISE 104	3213(B)	THY 3213	LRP2	male?	20-30				
RISE 105	cranium 1165 x20 (locality-ID: 110612-56)	THY 1165	Lower molar	male?	30-35				
RISE 106	grave A (x13B)	THY 1550 x13B	LRM1	male?	25-35			Gracile, long slender skeleton.	Shoveling incisors and talon cusps, maxillary second premolars with three roots.
RISE 107	N1 og N3	Thy 1007	Um1	nd	subadult				
RISE 108	Thy 1417x41, grave N2, (fibula period III) central grave	Thy 1417 x41, NM I 2858/79	URP2	nd	13-15				Slight shoveling of URI2 and ULI2.
RISE 166	grave A 38, per. II?. Central grave	NÆM 1997:103	ULP1	male?	25-35	45		Spina bifida	Abscess above ULI1. Significant dental wear on incisors, less on molars. Slight shoveling of URI2 and ULI2. Small teeth.
RISE 167	grave A 35	NÆM 1997:103	ULI1	male?	40+				Slight shoveling of URI2 and ULI2. Talon cusps on ULC. Small teeth.
RISE 168	grave A 37, per. II	NÆM 1997:103	premolar	male?	30-40		170 cm (measured in gravee)		Slight shoveling of lower incisors. Agenesis of LRC. Small teeth.
RISE 169	grave C, x31-45, ca. 1500 BC	GIM 3161	LRP2	male	25-35		(181.5 cm)	Large and robust skeleton.	Shoveling of URI2 and ULI2. LEH on LRC and LLC. Dental roots are long.
RISE 170		FSM 8903-11	LRP2	male?	20-25			Long slender long bones.	LEH on canines. Calculus. Wide mandible and relatively large teeth.
RISE 273		NM 271/45	ULP2	female	20-35			Perimortem blunt force trauma on right parietal.	
RISE 274		NM 771/41	URC	male	35-45+				Calculus.
RISE 275		NM 446/47, 765/47	LLP1	male	25-35			Ossicles at lambda.	Calculus, LEH (two bands on mid crown) on LRC and LLC and URP1.
RISE 276		NM 132/16, 784/51	LRP1	male	40-60				Heavy tooth wear.
RISE 281		NMB 10425	molar	nd	*c.* 15				
RISE 282		NMB 11022	URM1	nd	*c* . 5				
RISE 326	"Nybølmanden" PMD 264	Haderslev Museum	premolar	male	25-35		(170 cm)	Pronounced muscle attachments on right humerus.	Slight calculus. The ULC is retained (impacted), though fully developed and visible in palate.

RISE 433	x1	FSM 6714	LLC	male	45-60	Metopic suture.	Severe caries on LRM2 with abscess. Moderate calculus and heavy tooth wear in particular on ULM1 and LRM1.
RISE 460		Skive Museum 1974	URP1	nd	30-35		Caries. Abscess. Slight shoveling of incisors and thick enamel.

AM = ante mortem, LEH = Linear Enamel Hypoplasia. * Stature was previously calculated using Trotter and Gleser (1958), however there is no consistency in element that was used.
