# Supplementary material for: Mapping human mobility during the third and second millennia BC in present-day Denmark
Source: PLoS One. 2019 Aug 21;14(8):e0219850. doi: 10.1371/journal.pone.0219850 (PMC6703675; doi:10.1371/journal.pone.0219850)
Supplement: S2 Table — (PDF) [file pone.0219850.s003.pdf]

S3 Table. Crude prevalence of the most common skeletal and dental pathologies observed among the 88 individuals.

		Male	Female	Adult	Subadult	**TOTAL**
**Dental pathology**						
Caries		4	2	2		8
Calculus		12	7	2	1	22
Periodontitis		5	2			7
Abscess		4	1	1		6
LEH (Linear Enamel Hypoplasia)		13	4	1		18
**Skeletal pathology**						
Congenital		3				3
Joint disease	Degenerative joint disease -vertebrae	2				2
	Degenerative joint disease- joints					
	Osteoarthritis - vertebrae	1				1
	Osteoarthritis - joints	1				1
	Non specific infection - long bones	1				1
Miscellaneous	Cribra Orbitalia	4	1		2	7
	Porotic Hyperostosis	1	1			2
	Porosity in palate	3				3
Trauma		1	2			3
**Skeletal or dental other**						
Long slender bones		4	1	1		6
Secondary dental non-metric traits		16	6	3	2	27
Toothpick furrow		2				2
